# The role of PQL genes in response to salinity tolerance in *Arabidopsis* and barley

**DOI:** 10.1002/pld3.301

**Published:** 2021-02-10

**Authors:** Mashael Alqahtani, Damien J. Lightfoot, Fouad Lemtiri‐Chlieh, Ebtihaj Bukhari, José M. Pardo, Magdalena M. Julkowska, Mark Tester

**Affiliations:** ^1^ Division of Biological and Environmental Sciences and Engineering King Abdullah University of Science and Technology Thuwal Kingdom of Saudi Arabia; ^2^ Biology Department Princess Nourah Bint Abdul Rahman University Riyadh Kingdom of Saudi Arabia; ^3^ Department of Neuroscience University of Connecticut School of Medicine Farmington CT USA; ^4^ Instituto de Bioquimica Vegetal y Fotosintesis (IBVF) Consejo Superior de Investigaciones Científicas (CSIC) University of Seville Seville Spain

**Keywords:** arabidopsis, ion transport, PQL, salt stress

## Abstract

While soil salinity is a global problem, how salt enters plant root cells from the soil solution remains underexplored. Non‐selective cation channels (NSCCs) are suggested to be the major pathway for the entry of sodium ions (Na^+^), yet their genetic constituents remain unknown. Yeast PQ loop (PQL) proteins were previously proposed to encode NSCCs, but the role of PQLs in plants is unknown. The hypothesis tested in this research is that PQL proteins constitute NSCCs mediating some of the Na^+^ influx into the root, contributing to ion accumulation and the inhibition of growth in saline conditions. We identified plant PQL homologues, and studied the role of one clade of PQL genes in Arabidopsis and barley. Using heterologous expression of *AtPQL1a* and *HvPQL1* in HEK293 cells allowed us to resolve sizable inwardly directed currents permeable to monovalent cations such as Na^+^, K^+^, or Li^+^ upon membrane hyperpolarization. We observed that GFP‐tagged PQL proteins localized to intracellular membrane structures, both when transiently over‐expressed in tobacco leaf epidermis and in stable Arabidopsis transformants. Expression of *AtPQL1a*, *AtPQL1b,* and *AtPQL1c* was increased by salt stress in the shoot tissue compared to non‐stressed plants. Mutant lines with altered expression of *AtPQL1a, AtPQL1b,* and *AtPQL1c* developed larger rosettes in saline conditions, while altered levels of AtPQL1a severely reduced development of lateral roots in all conditions. This study provides the first step toward understanding the function of PQL proteins in plants and the role of NSCC in salinity tolerance.

## 1 INTRODUCTION

Soil salinization is a global issue that limits agricultural productivity, with more than 800 million hectares of the world's arable land being classified as salt‐affected (FAO, 2008). Salt‐affected land occurs predominantly in arid and semi‐arid regions due to poor irrigation practices or insufficient drainage (Rengasamy, 2002). The world population is projected to reach 9 billion by 2050 (Godfray et al., 2010) and, as such, more food will be required to feed an increasing human population. Since the area of arable land is limited, agricultural productivity (i.e., yield) must increase to meet future food demands. Increasing the yield of crops grown on saline soils, and the development of salt tolerant crops that can grow on marginal land that is currently considered unsuitable for agriculture, will help to meet these future requirements.

Responses of plants to salinity are classified into two phases: (a) early stress responses which are independent of shoot ion accumulation, the so‐called osmotic phase, and (b) the ionic phase, where plants experience the toxic accumulation of ions in the shoot (reviewed by Munns & Tester, 2008). Plants can reduce the detrimental effects experienced during the ionic phase by excluding sodium ions (Na^+^) from photosynthetically active tissues, compartmentalizing the ions into the non‐photosynthetically active tissues, and at the sub‐cellular level, to the vacuoles. Several families of ion transporters (e.g., HKT, SOS) have been demonstrated to be important for controlling Na^+^ and K^+^ movement within the plant (reviewed by Isayenkov & Maathuis, 2019; van Zelm et al., 2020). However, little is known about Na^+^ influx into the root cells from the soil solution (Roy et al., 2014). As this initial Na^+^ influx into plant roots is correlated with final Na^+^ accumulation in the shoot (Horie et al., 2012; Tester & Davenport, 2003), it is important to characterize the genetic components regulating Na^+^ influx into the roots.

The pathways proposed for Na^+^ influx into root cells comprise protein‐mediated Ca^2+^‐sensitive and insensitive pathways as well as the bypass flow (Tester & Davenport, 2003). K^+^ uptake transporters in the KUP family and high‐affinity K^+^ transporters in the HAK family are Ca^2+^ insensitive and have been demonstrated to facilitate transport of Na^+^ (Alemán et al., 2014; Bañuelos et al., 2002; Fulgenzi et al., 2008; Santa‐María et al., 1997; Takahashi et al., 2007). However, these transporters were shown to transport Na^+^ only in heterologous systems and appear to account for a small fraction of Na^+^ transport under conditions of low K^+^ and high Na^+^. The addition of extracellular Ca^2+^ alleviates the toxic effects of salt stress on plants by reducing Na^+^ influx (reviewed by Tester & Davenport, 2003), suggesting that Ca^2+^‐sensitive pathways play an important role in Na^+^ influx. Low Affinity Cation Transporter 1 (LCT1) was previously suggested to mediate the Ca^2+^ dependent influx of Na^+^, When LCT1 was expressed in yeast, it increased Na^+^ influx, and the Na^+^ influx was blocked after addition of Ca^2+^ to the external solution (Amtmann et al., 2001).

Non‐selective cation channels (NSCCs) were suggested as the major Ca^2+^‐sensitive pathway for Na^+^ influx in several species, including wheat (Davenport & Tester, 2000; Tyerman et al., 1997), barley (Amtmann et al., 1997), maize (Roberts & Tester, 1997), rye (White & Lemtiri‐Chlieh, 1995; White & Tester, 1992), and Arabidopsis (Demidchik & Tester, 2002; Essah et al., 2003). Electrophysiological characterizations revealed that the NSCCs which are responsible for the majority of Na^+^ influx are Ca^2+^‐sensitive and voltage‐insensitive NSCCs (vi‐NSCCs; Amtmann & Sanders, 1998; Davenport & Tester, 2000; Demidchik et al., 2002; Demidchik & Maathuis, 2007; White, 1999). However, the genes encoding Ca^2+^‐sensitive vi‐NSCC activity have not been identified. Previous electrophysiological studies demonstrated that Ca^2+^‐sensitive vi‐NSCCs show (a) weak discrimination between monovalent cations, with K^+^ favored over Na^+^, (b) a generalized selectivity sequence of K^+^ >NH_4_^+^ >Rb^+^ ≈ Cs^+^ ≈ Na^+^ >Li^+^ >TEA^+^, (c) inhibition by low external pH, and (d) inhibition by divalent cations such as Ca^2+^ (Demidchik & Tester, 2002).

In yeast, electrophysiological activities of a non‐specific cation channel 1 (NSC1) have been reported and this activity is similar to the Ca^2+^‐sensitive vi‐NSCC activity in plant roots (Bihler et al., 1998, 2002). Given the identification of Ca^2+^‐sensitive vi‐NSCC‐like activity in yeast, previous research (Carter, 2009; Tester et al., 2013) sought to identify candidate genes in yeast, as these could inform the search for plant Ca^2+^‐sensitive vi‐NSCC. Carter (2009) used an in silico screen to find possible candidate genes for vi‐NSCC activity in *Saccharomyces cerevisiae* and identified two candidates, *YOL092w* (*ScPQL1*) and *YDR352w* (*ScPQL2*). Bioinformatics analysis was performed and revealed that *ScPQL1* and *ScPQL2* are putative members of the PQ‐loop (PQL) protein family (Pfam PF04193; Carter, 2009; Tester et al., 2013). PQL protein family members are predicted to be membrane bound with five or seven transmembrane helices. Members of this family contain one or two conserved pairs of proline (P) and glutamine (Q) amino acids within a broader, weakly conserved region of 40–60 amino acids (Saudek, 2012). The physiological characteristics of ScPQL1 and ScPQL2 using heterologous expression in the *Xenopus laevis* oocyte system revealed that both proteins facilitate monovalent cation influx and were inhibited by low external pH and the presence of divalent cations (Carter, 2009; Tester et al., 2013). The transport activities of ScPQL1 and ScPQL2 are similar to NSC1 activity (Bihler et al., 1998, 2002), making them a putative candidate for NSCC.

In this manuscript we identified plant homologues of *ScPQL1* and *ScPQL2* and characterized the role of clade 1 PQLs in *Arabidopsis thaliana* and barley under control and salt stress conditions. Using electrophysiological studies in heterologous systems, we show that clade 1 PQLs facilitate transport of the monovalent cations, which is inhibited by external high Ca^2+^. While the studied mutants of clade 1 PQLs developed larger rosettes under salt stress conditions, we did not observe any changes in ion accumulation in the shoot. Intriguingly, the clade 1 PQL localized to the intracellular membrane structures, and co‐localized with tonoplast markers. We observed that alteration of *AtPQL1* levels severely affects lateral root development under non‐stress conditions. Our findings provide a first step toward understanding plant PQL function, indicating that clade 1 PQLs show similar transport properties to other Ca^2+^‐sensitive vi‐NSCCs, and play an important role in lateral root development.

## 2 MATERIALS AND METHODS

### 2.1 Sequence analysis

The sequences of two previously characterized *S. cerevisiae* PQ loop (PQL) proteins, ScPQL1 and ScPQL2 (Carter, 2009; Tester et al., 2013), were used to query the plant sequence database GeneBank (http://www.ncbi.nlm.nih.gov). Sequences were aligned using ClustalW (Thompson et al., 1994) with default values (gap opening penalty of 10 and gap extending penalty of 0.02). Sequences and alignments were assessed and edited with Jalview v2.0 (http://www.jalview.org/). Phylogenetic trees were constructed in Molecular Evolutionary Genetics Analysis Version 7 (MEGA7; Kumar et al., 2016) based on the amino acid sequences utilizing the Neighbor‐Joining tree building method (Saitou & Nei, 1987). A bootstrap value of 10,000 was used and the human PQL protein HsCTNS was set as the outgroup. The sequences used for the phylogenetic analysis were AtPQL1a (NP_193743), AtPQL1b (NP_850340), AtPQL1c (CAB16817), AtPQL2a (NP_200755), AtPQL2b (NP_567315), and AtPQL3 (NP_198883) from *A. thaliana*; MtPQL1a (XM_003618869.2), MtPQL1b (XM_003624798.2), MtPQL1c (XM_003611849.2), MtPQL1d (XM_003629456.2), MtPQL2 (XM_003589081.2), and MtPQL3 (XM_013587927.1) from *Medicago truncatula;* SlPQL1 (XP_004233648.1), SlPQL2 (XM_004252705.2), SlPQL3a (XM_010317644.1), SlPQL3b (XM_004231770.2), and SlPQL3c (XM_004233961.2) from *Solanum lycopersicum; Zm*PQL1 (NP_001141539), *Zm*PQL2 (ACR34772), and *Zm*PQL3 (NP_001149841) from *Zea mays*; OsPQL1 (NP_001042681), OsPQL2 (NP_001059633), and OsPQL3 (NP_001066568) from *Oryza sativa*; HvPQL1 (BAJ92450), HvPQL2 (BAJ85929), and HvPQL3 (BAJ95769) from *Hordeum vulgare*; HsCTNS (NP_004928) from *Homo sapiens*; and ScPQL1 (NP_014549) and ScPQL2 (NP_010639) from *S. cerevisiae*.

Protein topologies were predicted using the HMMTOP (Tusnády & Simon, 1998, 2001) and visualized using the TMRPres2D (Spyropoulos et al., 2004) for AtPQL1a (NP_193743), AtPQL1b (NP_850340), and AtPQL1c (CAB16817) from *A. thaliana*; OsPQL1 (NP_001042681) from *O. sativa*; HvPQL1 (BAJ92450) from *H. vulgare*; ScPQL1 (NP_014549) and ScPQL2 (NP_010639) from *S. cerevisiae;* and HsCTNS (NP_004928) from *H. sapiens*.

### 2.2 Heterologous protein expression in yeast

#### 2.2.1 Plasmid construction

The *AtPQL1a*, *AtPQL1b*, *AtPQL1c,* and *HvPQL1* cDNA were amplified from Arabidopsis Col‐0 cDNA and barley cDNA (cv. Morex) using standard PCR techniques (Green & Sambrook, 2012) with a proof‐reading polymerase KOD Hot Start DNA Polymerase (Novagen) and the relevant primers Table S1. Gel electrophoresis was used to verify the quality and quantity of the PCR products. The desired PCR products were extracted from the gel using the QIAEX^®^II Gel Extraction kit (QIAGEN) and transferred into the pCR8/GW/TOPO TA Gateway entry vector (Life Technologies), followed by transformation into *Escherichia coli* One Shot TOP10 Chemically Competent cells (Life Technologies). Plasmid DNA of positive clones was isolated and verified utilizing colony PCR (Green & Sambrook, 2012) and Sanger sequencing (Sanger et al., 1977). Subsequently, the *AtPQL1a*, *AtPQL1b*, *AtPQL1c,* and *HvPQL1* cDNA were transformed into the pAG426GAL‐ccdB‐EGFP Gateway Destination vector (a gift from Susan Lindquist (Addgene plasmid # 14203)) by LR recombination with Gateway cloning technology (Invitrogen). These constructs were confirmed by Sanger sequencing (Sanger et al., 1977).

#### 2.2.2 Yeast transformation

Two yeast strains were transformed with the above constructs. AXT3 is a salt‐sensitive strain derived from W303‐1B and has a genotype of *ena1‐4Δ::HIS3, nha1Δ::LEU2, nhx1Δ:: TRP1* (Venema et al., 2002). The second strain, LL178 that lacks the native *PQL1*, *PQL2,* and *PQL3* genes and is derived from the Σ1278b WT strain. The genotype of LL178 is *ura3 ypq1Δ ypq2Δ ypq3Δ* (Jézégou et al., 2012). LL178 was obtained from Dr. Bruno Gasnier and Dr. Bruno André. The yeast transformations were performed by the lithium acetate method according to Gietz and Woods (2002). The untransformed yeast strains were inoculated into 10 ml of yeast extract peptone dextrose YPD medium (1% (w/v) yeast extract, 2% (w/v) bactopeptone, 2% (w/v) glucose, and adenine 15 µg/ml) and incubated overnight at 30°C with 200 rpm shaking. On the next day, 2 ml of each culture was centrifuged at 13,000 rpm at room temperature for 30 s and the supernatant was removed. To the cell pellet, the transformation mixture was added (240 µl of PEG 3,350 (50%, w/v), 36 µl of Lithium acetate 1.0 M, 50 µl of Boiled SS‐Carrier DNA (2 mg/ml), and 34 µl Plasmid DNA (0.1 to 1 µg)). The mixtures were incubated in a water bath of 42°C for 45 min then centrifuged at 13,000 rpm at room temperature for 30 s and the supernatant was discarded. The cells were resuspended using 1 ml sterile water and 10 µl or 100 µl was spread onto plates with selective synthetic defined (*SD*) medium (0.17% yeast nitrogen base without amino acids and ammonium sulfate, 0.5% ammonium sulfate, 0,077% complete supplement mix (CSM) drop‐out‐Uracil (‐URA; Formedium), 2% glucose or galactose; 2% agar for solid media, and pH adjusted to 5.6 with KOH). The plates were incubated at 30°C for 3–4 days.

#### 2.2.3 Functional assay of PQLs on solid media

For salt growth assessment, three independent transformed yeast colonies per construct were inoculated into arginine phosphate AP medium (10 mM L‐arginine, 8 mM phosphoric acid, 2% glucose, 2 mM MgSO4, 1 mM KCl, 0.2 mM CaCl_2_, trace minerals and vitamins, 0.077% complete supplement mix (CSM) drop‐out‐Uracil (Formedium), 2% glucose or galactose; 2% agar for solid media and pH adjusted to 6.5 with L‐arginine; Rodríguez‐Navarro & Ramos, 1984) and incubated at 30°C overnight. Serial tenfold dilutions were made from the overnight culture starting at optical density (OD_600nm_ = 0.3–0.4). Spot assays were conducted on AP‐galactose plates supplemented with 2% galactose to induce the expression of *AtPQL1a*, *AtPQL1b*, *AtPQL1c*, *HvPQL1,* and *SbSaltol* (*SbSalt_tol2*; +ve). The positive control used was a salt‐tolerance protein (*SbSaltol*) from a proteomic study of *Salicornia bigelovii* conducted in our laboratory (Salazar, 2017). A range of salt stress was tested (30 mM NaCl, 35 mM NaCl, 50 mM KCl, and 1 mM LiCl) and the plates were incubated at 30°C for 3 days. The digital images were taken at the end of the experiment using a Canon digital camera.

#### 2.2.4 Functional assay of PQLs on liquid media

To quantify the effect of salt stress on the yeast growth, a functional assay of transformed yeast in liquid media has performed. Yeast colonies of *AtPQL1a*, *AtPQL1b*, *AtPQL1c*, *HvPQL1*, empty vector, or *SbSaltol* were used to inoculated 5 ml YPD‐glucose media supplemented with complete supplement mix (CSM) drop‐out‐Uracil in 50‐mL falcon tubes. Subsequently, the cultures were incubated at 30°C with shaking (200 rpm) for 2 days. To start the assay, 200 µl of the previous culture was used to inoculate 5 ml of YPD‐glucose and/or YPD‐galactose in 50 ml falcon tubes and incubated at 30°C with shaking (200 rpm) for 24 hr. The assay was conducted in Corning^®^ 48 flat‐bottom well plate using yeast culture with OD_600nm_ adjusted at 0.3–0.4 added to 250 µl *SD*‐glucose as a positive control and/or *SD*‐galactose supplemented with complete supplement mix (CSM) drop‐out‐Uracil in each well. For the treatments, the media were additionally supplemented with 35 mM NaCl, 50 mM NaCl, 50 mM KCl, or 1 mM LiCl. An automated plate reader (Varioskan® Flash) was set to record reads of the OD_600nm_ for each well every 30 min for 3 days.

### 2.3 Heterologous protein expression and electrophysiological characterization using HEK293 cells

The *AtPQL1a and HvPQL1* cDNA were cloned into mammalian expression vector pcDNA6.2‐EmGFP vector (Vivid ColorsTM pcDNATM6.2/EmGFP‐DEST) using the GeneArt^TM^ Gene Synthesis (ThermoFisher Scientific). Their expression was driven by T7 promoter. These constructs were used for human embryo kidney 293 (HEK293) cell line (Cat. no. R70007, Life Technologies, Carlsbad, CA) transfection performed according to (Ooi et al., 2016) protocol. HEK293 cells were maintained in Dulbecco's modified Eagle's medium DMEM (Thermo, 10569‐044) supplemented with 10% (v/v) fetal bovine serum FBS (Thermo, 10100147), and 1% (v/v) penicillin‐streptomycin (10,000 U/ml) in Cell Culture Flask T75 (Eppendorf, 0030711.122) at 37°C in a humidified atmosphere of 5% CO_2_. Prior to the transfection, poly‐D‐Lysine hydrobromide‐coated round coverslips were prepared. The round coverslips first soaked in acetone inside a fume hood overnight, then rinsed the coverslips by absolute ethanol and sterile water. The coverslips were placed in the oven 65°C overnight to dry. Subsequently, 50 μg/ml poly‐D‐Lysine hydrobromide was added to three clean coverslips that placed in 6‐well flat‐bottom cell culture plate and incubated at 37°C overnight. Finally, the coated coverslips were washed using sterile water. For the transient transfection, the 2.5 × 10^5^ viable HEK293 cells per 2 ml DMEM were added to the 6‐well plate that contains the coated coverslips. The lipid‐mediated DNA‐transfection procedure with LipofectAMINE was followed to introduce 2.5 μg of AtPQL1a or HvPQL1 DNA into cells for expression of protein. The 6‐well plate then incubated at 37°C overnight to allow the cells to attach to the coated coverslips and allow the proteins to express. In the following day, the AtPQL1a and HvPQL1 expressions were assessed using the green fluorescence filter (Nikon Eclipse TS100, Melville, NY) at the excitation wavelength from 460 to 500 nm. The positive green‐fluorescent cells were selected for patch clamp.

The patch clamp rig has an inverted microscope Carl Zeiss Axio Observer.A1 (Carl Zeiss, Oberkochen, Germany) to visualize the transfected cells. Recording pipettes were pulled from thick/standard wall borosilicate glass capillaries (B150‐86‐10, Sutter Instrument^®^) using a P‐1000 Flaming/Brown^TM^ micropipette puller (Sutter Instrument®, Novato, CA) and the resistance of the pipette was between 3 and 5 MΩ when filled with an intracellular solution consisting of 120 mM K‐Gluconate, 25 mM N‐methyl‐D‐glucamine, 2.5 mM NaCl, 1 mM CaCl_2_, 2 mM MgCl_2_, 2 mM ATP (K‐or Mg‐ salt), 10 mM EGTA, and 10 mM HEPES (pH 7.3 and Osmolality at 290 ± 5 mOsm.kg^−1^, adjusted by adding sorbitol). The external bath solution contained 10 mM KCl, 135 mM N‐methyl‐D‐glucamine, 0.05 mM CaCl_2_, 0.5 mM MgCl_2_, 10 mM HEPES, and 1 mM glucose (pH 7.3 and Osmolality at 310 ± 5 mOsm.kg^−1^, adjusted by adding sorbitol). In other experiments, KCl was replaced by 10 mM NaCl or 10 mM LiCl. Upon achieving whole‐cell configuration, the cells were maintained at a holding potential of −20 mV. The voltage‐clamp protocol consists of a series of 1‐s‐long squared voltage tests between +40 mV and −120 mV in 20 mV decrements to record the Na^+^, K^+^ and Li^+^ currents. The patch clamp experiments were conducted at room temperature. A MultiClamp^TM^ 700B microelectrode amplifier (Axon Instruments, Molecular Devices) was used. The pulse protocol, data acquisitions, and analysis were performed using pClamp software (ver. 10 package, Molecular Devices). The signals were low‐pass filtered at 2 kHz before analog‐to‐digital conversion and were uncorrected for leakage current or capacitive transients. The data were expressed as mean ± standard error of the mean.

### 2.4 Transient co‐localization of PQLs in Arabidopsis and barley with tonoplast marker

The co‐localization of AtPQL1a, AtPQL1b, AtPQL1c, and HvPQL1 with tonoplast marker determined using a transient assay with *Nicotiana benthamiana* epidermal cells. The coding sequence (CDS) of AtPQL1a, AtPQL1b, AtPQL1c, and HvPQL1 was amplified from Col‐0 cDNA using primers (Table S1) spanning the CDS without stop codon by PCR with Phusion High‐Fidelity DNA Polymerase (New England Biolabs). Amplicons were then purified and cloned into Gateway entry vector pENTR‐D/Topo (Invitrogen) to generate entry vector (without stop codon). Followed by cloning into the Gateway expression destination vector pUBC‐GFP‐Dest vector (Grefen et al., 2010) by LR reaction following the manufacturer's instructions (Invitrogen). The UBQ10p::AtPQL1a::eGFP, UBQ10p::AtPQL1b::eGFP, UBQ10p::AtPQL1c::eGFP, UBQ10p::HvPQL1::eGFP, and the tonoplast marker (CD3‐975::*mCherry*; Nelson et al., 2007) were transformed into *Agrobacterium tumefaciens* strain GV3101 (Koncz & Schell, 1986) by the heat‐shock protocol (Sparkes et al., 2006). The overnight culture of transformed *Agrobacterium* constructs as well as P19, a viral gene silencing suppressor (Voinnet et al., 2003), inoculated into 10 ml LB media with appropriate antibiotics, and incubated for 24 hr at 28°C with agitation. The following day, each culture was centrifuged at 5000 *g* for 15 min and resuspend using the infiltration buffer (10 mM MgCl2, 10 mM MES pH 5.7, 150 μM acetosyringone) and stored in the dark for 2–3 hr. The final OD_600nm_ was adjusted to 0.4. Three‐week‐old *N. benthamiana* plants were infiltrated with UBQ10p::AtPQL1a::eGFP, UBQ10p::AtPQL1b::eGFP, UBQ10p::AtPQL1c::eGFP, and UBQ10p::HvPQL1::eGFP along with tonoplast marker (CD3‐975::*mCherry*; Nelson et al., 2007).

The co‐localization was visualized 72 hr after infiltration. Image was captured using confocal laser scanning microscope (LSM‐880, Zeiss). Images were analyzed with Fiji image analysis software (Schindelin et al., 2012). The eGFP excitation was at 488 nm and the emission was between 505 and 530 nm, while the excitation for *mCherry* was at 515 nm and the emission was at 734 nm.

### 2.5 Stable transformation of AtPQL1b and subcellular localization assay

The UBQ10p::AtPQL1b::eGFP was used for floral dip transformation (Zhang et al., 2006) in order to generate transgenic Arabidopsis overexpression stable line in Col‐0 background. Transgenic plants were selected on 0.5 MS medium containing 10 μg/mL BASTA. Eight‐day‐old plantlet roots of stable transgenic T3 homozygous lines of Arabidopsis plants overexpressing UBQ10p::AtPQL1b::GFP were stained by Propidium Iodide following (Helariutta et al., 2000) protocol. In order to visualize the GFP and Propidium Iodide fluorescence signals, images were captured with Zen 2.3 image software (Zeiss, Germany) using confocal laser scanning microscope (LSM‐880, Zeiss, Germany). Images were processed with Fiji image analysis software (Schindelin et al., 2012). The eGFP excitation was at 488 nm and the emission was at 505–530 nm, while the excitation for Propidium Iodide dye was 515 nm and the emission was between 615–734 nm.

### 2.6 Native expression of PQLs in Arabidopsis and barley using qRT‐PCR

#### 2.6.1 Collecting tissue from Arabidopsis for expression analysis

Seeds of Arabidopsis Columbia‐0 (Col‐0) wild type from our laboratory were used. The seeds were placed in seed holders (Araponics http://www.araponics.com/) filled with germination medium (Macronutrients (1 M CaCl_2_, 1 M KCl, 0.4 M Ca(NO_3_)_2_•4H_2_O, 0.4 M MgSO_4_•7H_2_O, 0.1 M KH_2_PO_4_) and Micronutrients (50 mM NaFe(III)EDTA, 50 mM H_3_BO_3_, 5mM MnCl_2_•4H_2_O, 10 mM ZnSO_4_•7H_2_O, 0.5 mM CuSO_4_•5H_2_O, 0.1 mM Na_2_MoO_3_)), 0.7% agar, and pH adjusted with KOH to 5.6 (Conn et al., 2013). Up to three seeds were placed onto the center of each seed holder filled with the germination medium. The seed holders were subsequently placed into the grid that forms the top part of 1 ml pipette tip boxes filled with germination medium. The boxes were covered with cling film to maintain high humidity and were put at 4°C in the dark for 2 days to break seed dormancy. After 2 days, boxes were transferred to the Conviron growth chamber set at 22°C temperature, 60% humidity, and 8 hr light/16 hr dark cycle. The lids were lifted gradually over the next 2 days to ensure gentle change in humidity. The germination medium was incrementally changed when the root fully emerged from the seed holder to basal nutrient solution (Macronutrients: 1 M NH_4_NO_3_, 1 M KNO_3_, 1 M CaCl_2_, 1 M KCl, 0.4 M Ca(NO_3_)_2_•4H_2_O, 0.4 M MgSO_4_•7H_2_O, 0.1 M KH_2_PO_4_, 1 M NaCl and Micronutrients: 50 mM NaFe(III)EDTA, 50 mM H_3_BO_3_, 5 mM MnCl_2_•4H_2_O, 10 mM ZnSO_4_•7H_2_O, 0.5 mM CuSO_4_•5H_2_O, 0.1 mM Na_2_MoO_3_) pH with KOH to 5.6 and 0.7% agar (Conn et al., 2013). Two weeks after germination, the Arabidopsis seedlings were transferred to an aerated hydroponics system (20 L per container, 35 plants per container) and were left to adapt to the new system for 1 week before the application of treatments (Conn et al., 2013). The seedlings were transferred to three different aerated hydroponic tanks which contained basal nutrient solution with or without supplementation of salt (100 mM of NaCl) added in 25 mM NaCl increments over 2 days or nutrient starvation treatment (no basal nutrient solution, 0.1 mM CaCl_2_). The shoot and root tissues were harvested after 1, 4, and 7 days after complete application of treatment.

#### 2.6.2 Collecting tissue from barley for expression analysis

Wild‐type barley (cv. Morex) seeds were obtained from Dr. Nils Stein (Leibniz Institute of Plant Genetics and Crop Plant Research). Seeds were sterilized in 70% ethanol for 5 min, and then washed three times with sterile water. Sterilized seeds were placed in petri dishes (100 × 25 mm) with moistened (milliQ water) filter paper (Whatman size 4). The petri dishes were covered with its lid and incubated for 4 days at room temperature. The germinated plantlets were transferred into a flood‐drain supported hydroponics system that contains general growth solution (Macronutrients (0.2 mM NH_4_NO_3_, 5 mM KNO_3_, 2 mM Ca(NO_3_)_2_•4H_2_O, 2 mM MgSO_4_•7H_2_O, 0.1 mM KH_2_PO_4_, 0.05 mM NaFe(III)EDTA) and Micronutrients (50 µM H_3_BO_3_, 5 µM MnCl_2_•4H_2_O, 10 µM ZnSO_4_•7H_2_O, 0.5 µM CuSO_4_•7H_2_O, 0.1 µM Na_2_MoO_4_•2H_2_O)) with 30 min cycles (Shavrukov et al., 2012). The plants were adapted to the new system for 10 days before starting the treatments application. The experimental treatments were general growth solution with or without salt stress (reaching 150 mM NaCl via three 50 mM NaCl increments over 24 hr) or nutrient starvation (no general growth solution, 0.1 mM CaCl_2_) that applied when the third leaf emerged (approximately 14 days after germination). The harvest time points were 1 and 10 days after complete treatments application. Whole root, third leaf blade, and third leaf sheath were harvested for further analysis.

#### 2.6.3 RNA extraction and qRT‐PCR analysis

RNA extraction was performed using Direct‐zol^TM^ RNA MiniPrep Plus Kit (Zymo Research, R2072) and first‐strand complementary DNA was synthesized from 1 μg of total RNA using reverse transcriptase, SuperScript® III First‐Strand Synthesis SuperMix kit (Invitrogen, 18,080,400). Quantitative RT‐PCR (qRT‐PCR) was performed using SYBR™ Select Master Mix (Applied Biosystems™) and BIO‐RAD CFX96 Real‐Time System instrument. The expression of At3g18780 (*AtActin2*) was used as a reference gene for normalization of gene expression levels in all Arabidopsis samples. The transcript levels of AK252297 (elongation factor‐1α (*HvEF1‐α*)) and U40042 (α‐TubulinA (*HvTubA*)) were used as reference genes for normalization of gene expression levels in all barley samples. The relative expressions of target genes were calculated by ∆Ct = 2^−Ct‐value target^⁄2^−Ct‐value reference^. For barley, the geometrical means of two normalized transcripts (*HvEF1‐α* and *HvTubA*) were calculated that represent the normalized expression of HvPQL1. Three biological and two technical replicates were used in the expression level determination. Primers used for this experiment are listed in Table S2.

### 2.7 High‐throughput phenotyping of salt stress responses

To study natural variation in the Arabidopsis transgenic materials in response to salt, Photon Systems Instrument (PSI) was used to perform high‐throughput phenotyping experiment. The plant materials used were Col‐0 as a control, constitutive overexpression lines (35S), and knockdown lines (amiRNA; Shearer, 2013). T‐DNA mutant lines in Col‐0 background for *AtPQL1a* (*Atpql1a* – SALK_108796), *AtPQL1b* (*Atpql1b‐1* SALK_001485 and *Atpql1b‐2* SALK_129118), and *AtPQL1c* (*Atpql1c‐1* – Salk_036418, *Atpql1c‐2* – Salk_044346, and *Atpql1c‐3* – Salk_060084) were ordered from NASC. All lines were examined for homozygous T‐DNA insertions using the primers designed with T‐DNA express. The seeds were germinated and grown as described in Awlia et al. (2016). Seeds were stratified in water and stored in 4ºC for 3 days in the dark. Subsequently, seeds were sown into pots (70 mm x 70 mm x 65 mm) filled with 85 g of fresh soil that was then watered to full water‐holding capacity. Fifteen replicates for each genotype were grown in controlled growth chamber (12 hr/ 12 hr light/dark cycle at 22°C and a relative humidity of 60%). The plants were weighed and watered automatically every day. Salt application occurred at 10‐leaf stage that was 17 days after germination. The salt applied by soaking the trays in 200 mM NaCl or water for 1 hr to achieve fully saturation stage for the soil. Subsequently, drained the pots for 10 min before placing it back to the PSI chamber. The actual concentration of the salt stress that plant exposed to was approximately 80 mM NaCl. The plants were automatically photographed from above every 12 hr during the day and night (2 p.m. and 2 a.m.) for 7 days. Each measuring round consisted of 15 min dark adaptation period inside the acclimation chamber then kinetic chlorophyll fluorescence (ChlF) and RGB imaging, weighing, and watering. Pixel count, color, and chlorophyll intensity were evaluated from images. The ChlF imaging was optimized using light curve protocol (Henley, 1993). The harvesting occurred after 7 days of salt applications which was the end of the experiment. The harvested whole shoot used to measure fresh and dry mass and Na^+^ and K^+^ accumulation using inductively coupled plasma‐optical emission spectrometry (ICP‐OES). Water content was calculated from the differences between the fresh and dry mass. Also, Na^+^/K^+^ ratio was calculated from the Na^+^ content over the K^+^ content per dry mass of the plant (Negrão et al., 2016). From the RGB images, the individual plant growth rate (GR) under salt stress was estimated by fitting exponential functions (Area = eDelta × time(days) + intercept) to the increase in the rosette area over the time from day 0 to 7 after salt stress application. Also, average growth rate of Projected Rosette Area at the end of the experiment relative to growth rate of Projected Rosette Area at the beginning of the salt application were calculated for both delta and intercept values. In addition, we calculated relative salt stress performance of each studied genotype by dividing growth rate of salt by the average growth rate at control conditions which called shoot‐ion independent tolerance (SIIT; Roy et al., 2014). The analysis of the effect of salt stress on all genotypes and treatments for the RGB traits were done using R studio software (Rstudio team, 2015) and statistically significant differences between the genotypes were determined using student's *t*‐test.

### 2.8 Root system architecture (RSA) assays

Col‐0 was used as a control, constitutive overexpression lines (35S), and knockdown lines (amiRNA; Shearer, 2013). T‐DNA mutant lines in Col‐0 background for AtPQL1a (*Atpql1a* – SALK_108796), AtPQL1b (*Atpql1b‐1* SALK_001485 and *Atpql1b‐2* SALK_129118), and AtPQL1c (*Atpql1c‐1* – Salk_036418 and *Atpql1c‐3* – Salk_060084) were ordered from NASC and/or ABRC. The seeds were surface sterilized with 20% (v/v) bleach for 10 min, then washed 5 times with sterile MilliQ water. Seeds were suspended in 0.1% agar and stratified in the dark at 4°C for 3 days. The seeds were sown on square petri dishes (12 × 12 cm) containing 40 ml of a growth medium (0.5 Murashige and Skoog (MS) medium, 0.5% sucrose, 0.1% M.E.S. monohydrate, and 1% Daishin agar, pH adjusted to 5.8 with KOH). Plates were placed vertically under long day conditions (22°C, 60% humidity, 16/8 hr light/dark cycle). Four days after germination, seedlings were transferred to square petri dishes containing 0.5 MS medium supplemented with 0, 75 or 125 mM NaCl, 125 mM KCl or 20 mM LiCl. The plates were scanned with Epson perfection V800 scanner at 200 dpi (dots per inches) every 2 days after transfer to the treatment plates. The main roots and lateral roots were measured at the sixth day after transfer using SmartRoot tool in ImageJ software (Lobet et al., 2011). The RSA traits included main root length (MRL), total lateral root length (TLRL), lateral root number (#LR), and total root length (TRL). For each experiment, 12 biological replicates per genotype and per condition were scored. The experiment was repeated three times with similar results. Statistically significant differences between the genotypes were determined using student's *t*‐test.

## 3 RESULTS

### 3.1 Identification of plant PQL proteins through phylogenetic relationships

**FIGURE 1:**
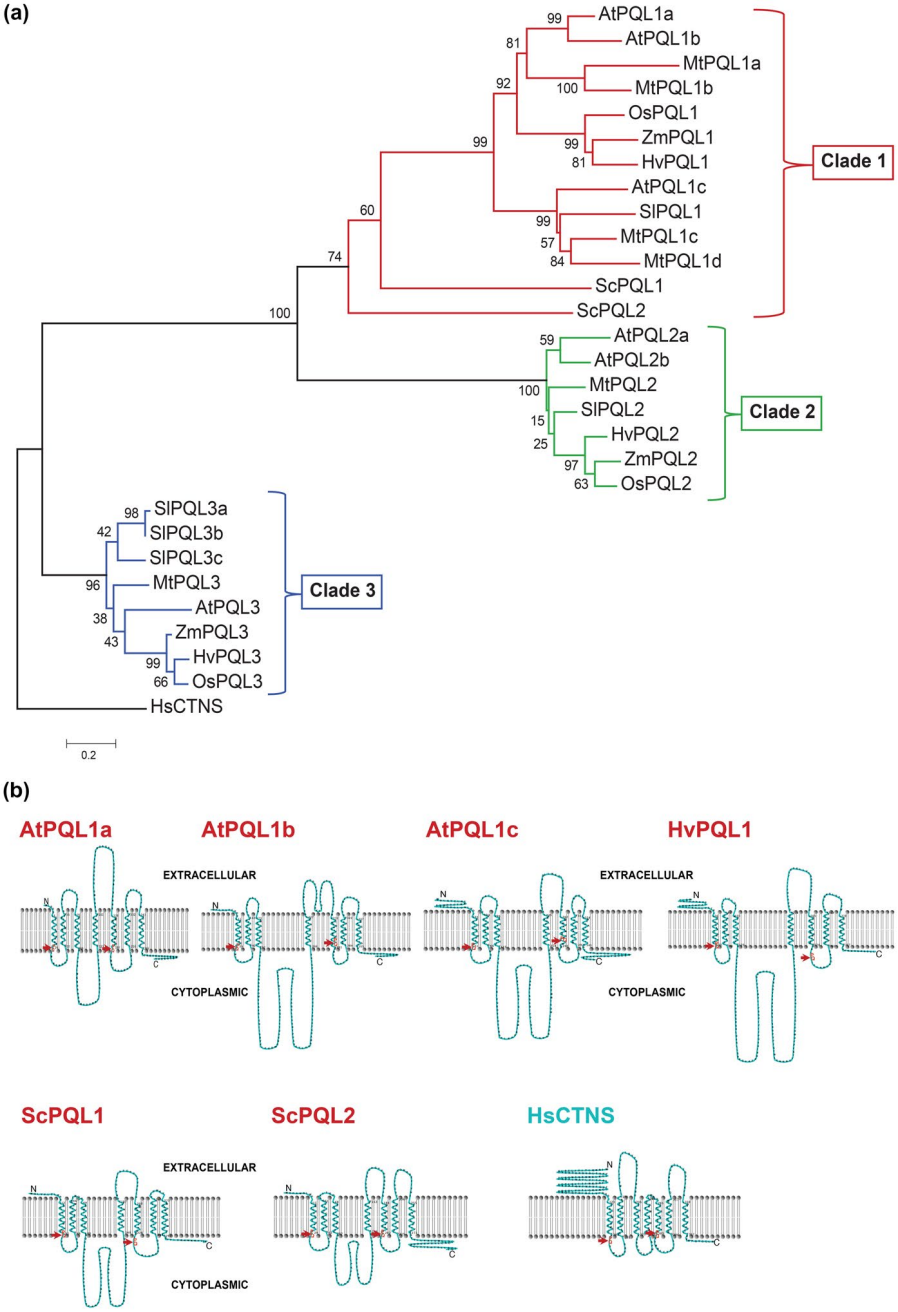
Identification of genes encoding PQL proteins in plants. (a) Sequence conservation between yeast and plant PQLs. A phylogenetic tree of yeast PQL protein sequences (ScPQL1 and ScPQL2), *Arabidopsis thaliana* (AtPQL1a, AtPQL1b, and AtPQL1c), *Medicago truncatula* (MtPQL1a, MtPQL1b, MtPQL1c, MtPQL1d, MtPQL2, and MtPQL3), *Solanum lycopersicum* (SlPQL1, SlPQL2, SlPQL3a, SlPQL3b, and SlPQL3c), *Zea mays* (ZmPQL1, ZmPQL2, and ZmPQL3), *Oryza sativa* (OsPQL1, OsPQL2, and OsPQL3), and *Hordeum vulgare* (HvPQL1, HvPQL2, and HvPQL3). *Homo sapiens* PQL protein, HsCTNS, was used as the outgroup. The tree was constructed in Molecular Evolutionary Genetics Analysis Version 7 (MEGA7) based on the protein sequences (Kumar et al., 2016) utilizing the Neighbor‐Joining tree building method (Saitou & Nei, 1987). Numbers on the nodes indicate bootstrap values as a percentage. Red box indicates clade 1, green box indicates clade 2, and blue box indicates clade 3 of PQL proteins. Scale bar indicates the number of amino acid substitutions per site. (b) Predicted protein topologies of PQLs belonging to clade 1. The protein transmembrane structures are predicted using the HMMTOP (Tusnády & Simon, 1998, 2001) and visualized using the TMRPres2D (Spyropoulos et al., 2004) with default settings. The topology of PQLs in clade 1 is predicted to have seven transmembrane domains and two PQ motifs, indicated by the red arrows. The part above and below the lipid bilayer indicates the extracellular and intracellular loop, respectively

We identified plant homologues of ScPQLs by performing a BLAST search on the NCBI database, using the full‐length cDNA sequences of *ScPQL1* and *ScPQL2*. A phylogenetic tree was constructed with PQL sequences from *Arabidopsis thaliana*, barley (*Hordeum vulgare*), rice (*Oryza sativa*), *Medicago truncatula*, maize (*Zea mays*), and tomato (*Solanum lycopersicum*). The tree contains three distinct clades (Figure 1a), where both yeast PQLs (*ScPQL1* and *ScPQLs*) group closest to clade 1 PQLs. Interestingly, there appears to be a difference between the size of the PQL gene families in monocot and dicot plants. Monocots (barley and maize) have one member in each clade, while dicot species (Arabidopsis, Medicago, and tomato) show multiple genes in each clade, except for clade 3. Given the highest similarity between the yeast PQLs and the clade 1 plant PQLs, *AtPQL1a*, *AtPQL1b*, *AtPQL1c,* and *HvPQL1* were selected for further investigation.

To examine further the protein similarity among PQLs in clade 1, we investigated the predicted protein secondary structures (Figure 1b). According to the predicted topologies, all PQL clade 1 members contain seven transmembrane domains **(**Figure 1b), resulting in three extracellular and three cytoplasmic loops. Members of clade 1 possess a relatively large second cytoplasmic loop and two PQ motifs (a doublet of proline (P) and glutamine (Q) amino acids). The first PQ motif is predicted to be located near the cytoplasmic side of the first transmembrane domain in all PQLs belonging to clade 1. However, the position of the second PQ motif is more variable, being either close to the cytoplasmic side following the fifth transmembrane domain (AtPQL1a, ScPQL2, Figure 1b), within the fifth transmembrane domain (AtPQL1b, AtPQL1c), or in the third cytoplasmic loop linking transmembrane domains 4 and 5 (ScPQL1, OsPQL1, HvPQL1, Figure 1b). Human PQL, HsCTNS, also has seven transmembrane domains and two PQ motifs. While the functional relevance of these features is unknown, the structural features, including number of transmembrane domains and location of first PQ motif, appear to be conserved among yeast and plant clade 1 members.

### 3.2 AtPQL1a, AtPQL1b, AtPQL1c, and HvPQL1 transport Na^+^, K^+^, and/or Li^+^ in yeast

**FIGURE 2:**
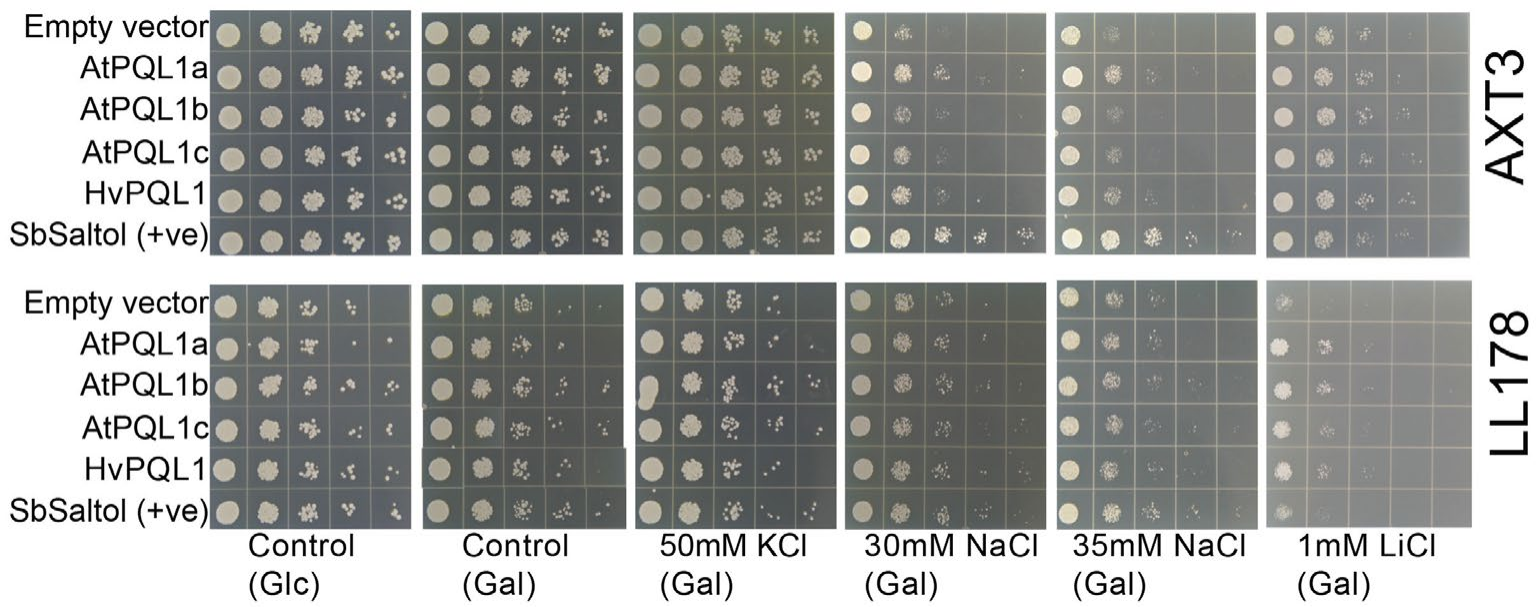
AtPQL1a and HvPQL1 promotes growth under saline conditions of salt‐sensitive yeast strain. *S. cerevisiae* salt‐sensitive strain (AXT3, upper panel) or strain lacking endogenous PQLs *ypq1‐3△* (LL178, lower panel) was transformed with the vectors containing *AtPQL1a*, *AtPQL1b*, *AtPQL1c*, *HvPQL1,* and *SbSaltol* (positive control) under the control of galactose inducible promoter. Empty Vector transformations were used as a negative control. The individual strains were grown in AP media containing glucose (Glc) or galactose (Gal), with or without supplementation of 50 mM KCl, 30 mM NaCl, 35 mM NaCl, or 1 mM LiCl to examine the strain tolerance to specific ions

The transport properties of AtPQL1a, AtPQL1b, AtPQL1c, and HvPQL1 were initially examined using yeast growth assays. The AXT3 and LL178 strains were transformed with constructs expressing *AtPQL1a*, *AtPQL1b*, *AtPQL1c*, *HvPQL1*, *SbSaltol* (+ve control,) or empty vector under the *GAL1* (Galactose) inducible promoter. Three independent yeast transformants per construct were plated at decreasing dilutions onto media containing different concentrations of NaCl, KCl, and LiCl (Figure 2). As with the yeast and mammalian PQL proteins (Jézégou et al., 2012), plant PQLs were localized in the yeast vacuole (Figure S1). In order to examine whether the expression of PQL genes affected yeast growth under optimal conditions, they were grown on the control media containing either glucose or galactose, or media supplemented with 50 mM KCl. All PQL constructs transformed into AXT3 or LL178 strains grew equally well under control conditions (Figure 2), indicating that the expression of PQL is neither crucial nor detrimental for yeast survival under non‐stress conditions.

Supplementation of 30 or 35 mM NaCl reduced yeast growth equally for all the constructs in LL178 compared to control conditions (Figure 2). However, when transformed LL178 strains were grown on media supplemented with 1 mM LiCl, the strains expressing Arabidopsis and barley PQLs (*AtPQL1a*, *AtPQL1b*, *AtPQL1c,* and *HvPQL1*) showed higher growth compared to strains transformed with empty vector or *SbSaltol*. The results obtained using the LL172 strain, which lacks the native yeast PQL genes, indicate that *AtPQL1a*, *AtPQL1b*, *AtPQL1c,* and *HvPQL1* confer increased Li^+^ tolerance but do not affect Na^+^ tolerance in yeast (Figure 2). However, strain LL172 maintains intact the ENA Na^+^‐pumps and the Na^+^,K^+^/H^+^ exchanger NHA1 acting at the plasma membrane, which might have cancelled the phenotypic effect of Na^+^ transport by plant PQLs at the yeast vacuole. For the salt‐sensitive yeast AXT3, which lacks these endogenous Na^+^ transporters, the strains transformed with *AtPQL1a* or *HvPQL1* grew slightly better in the media containing 30 or 35 mM NaCl compared to other PQL constructs and the empty vector (Figure 2). However, the growth of all strains was similar on media supplemented with 1 mM LiCl (Figure 2). The results obtained from salt‐sensitive AXT3 strain indicate that AtPQL1a and HvPQL1 might be involved in transport of Na^+^.

### 3.3 AtPQL1a and HvPQL1 showed permeability to monovalent ions such as Na^+^, K^+^ and/or Li^+^

**FIGURE 3:**
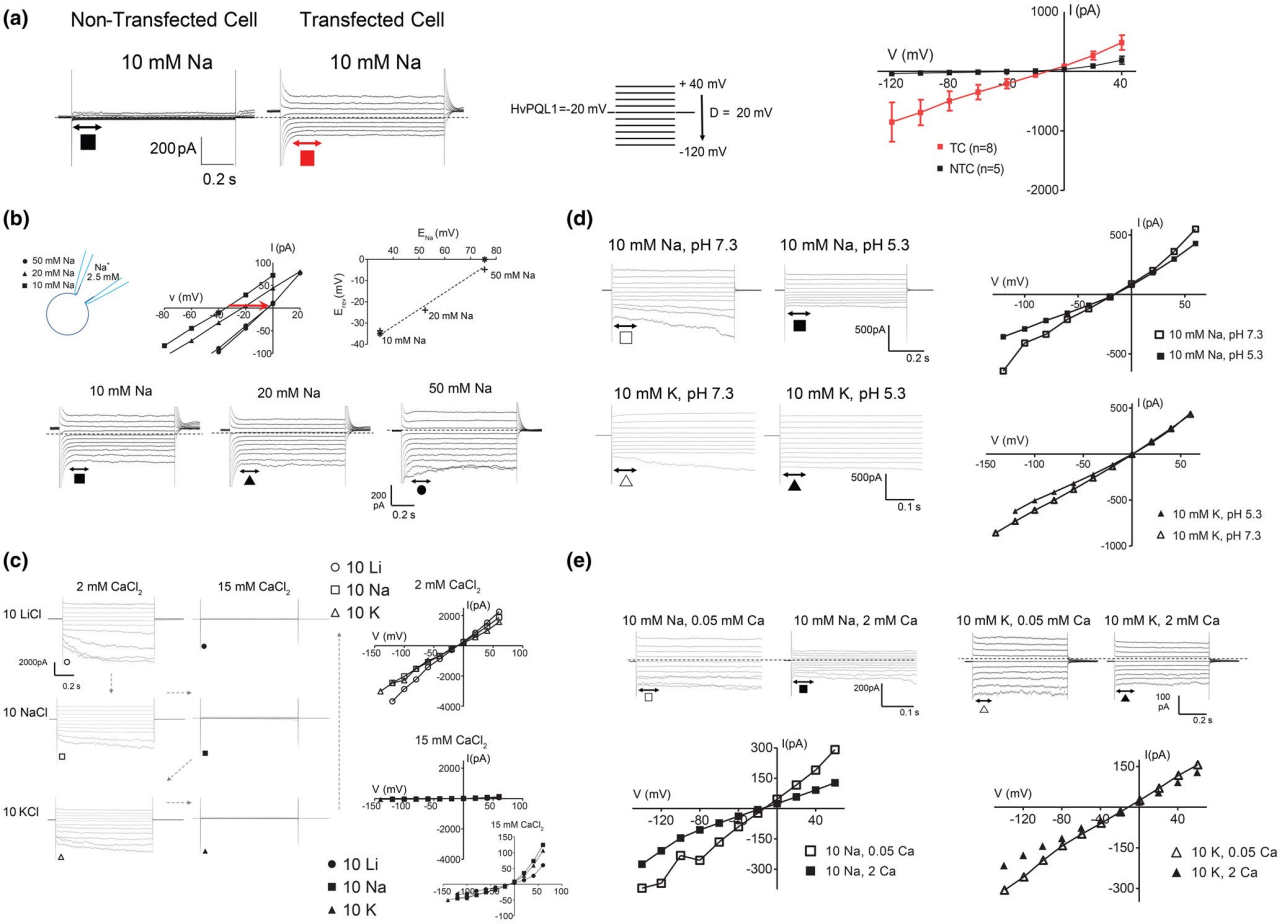
Electrophysiological characterization of HvPQL1 and AtPQL1a (a) Currents in whole‐cell configuration recorded from HEK293 cells transfected with *pcDNA6.2‐HvPQL1‐EmGFP* vector (TC, red) and non‐transfected (NTC, black) cells in 10 mM Na^+^ external solution (left panels, voltage protocol shown below the traces) and the I‐V plot (right panel) showing the average currents from NTC (5 cells) and TC (8 cells) in 10 mM Na^+^ external solution. (b) The sketch of the setup used, with the internal solution containing 2.5 mM Na^+^ while the external solutions contained three different Na^+^ concentrations 50 mM Na^+^ (●), 20 mM Na^+^ (▲), and 10 mM Na^+^ (■). Lower panels represent currents in whole cell configuration recorded from HEK293 cells transfected with HvPQL1 in 10 mM (■), 20 mM (▲), and 50 mM (●) external Na^+^. Middle upper panel represents I‐V plot indicating the shift in the reversal potential for Na^+^ (see red arrow), while upper right panel is E_Na_/E_rev_ relationship indicating that the voltage shift in E_rev_ is consistent with increasing Na^+^ concentrations. (c) Left panels represent currents in whole‐cell configuration recorded from HEK293 cells transfected with *HvPQL1* in 10 mM LiCl (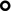), 10 mM NaCl (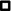), and 10 mM KCl (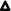) in presence of low (2 mM) concentration of CaCl_2_ in the external solutions. Middle panels represent currents in whole‐cell configuration recorded from HEK293 cells transfected with *HvPQL1* in 10 mM LiCl (●), 10 mM NaCl (■), and 10 mM KCl (▲) in presence of high (15 mM) concentration of CaCl_2_ in the external solutions. Upper right panel represent I‐V plot of transfected HEK293 with *HvPQL1* in 10 mM LiCl (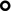), 10 mM NaCl (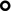), and 10 mM KCl (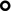) external solutions and in presence of low (2 mM) CaCl_2_, while lower right panel represents an I‐V plot of transfected HEK293 with *HvPQL1* in 10 mM LiCl (●),10 mM NaCl (■), and 10 mM KCl (▲) external solutions and in presence of high (15 mM) CaCl_2_ (inset: zoomed I/V plot in 15 mM external Ca^2+^ to highlight the persistence of *I*_A_‐like current). (d) Upper left and middle panels represent currents in whole‐cell configuration recorded from HEK293 cells transfected with *HvPQL1* in 10 mM NaCl, pH 7.3, and 10 mM NaCl, pH 5.3. Upper right panel represent an I‐V plot of transfected HEK293 with *HvPQL1* in 10 mM NaCl either in pH 7.3 (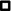) or pH 5.3 (■). Lower left and middle panels represent currents in whole cell configuration recorded from HEK293 cells transfected with *HvPQL1* in 10 mM KCl, pH 7.3, and 10 mM KCl, pH 5.3, while lower right panel represent an I‐V plot of transfected HEK293 with *HvPQL1* in 10 mM KCl either in pH 7.3 (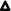) or pH 5.3 (▲). (e) Two upper left panels represent currents in whole‐cell configuration recorded from HEK293 cells transfected with *AtPQL1a* in 10 mM Na^+^ and in presence of 0.05 or 2 mM Ca^2+^ in the external solution, while lower left panel represents I‐V plot of transfected HEK293 with *AtPQL1a* in 10 mM Na^+^ and 0.05m Ca^2+^ (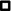) or 10 mM Na^+^ and 2 mM Ca^2+^ (■). Two upper right panels represent currents in whole‐cell configuration recorded from HEK293 cells transfected with AtPQL1a in 10 mM K^+^ and in presence of 0.05 (left panel) or 2 mM Ca^2+^ in the external solution (right panel), while lower right panel represents I‐V plot of transfected HEK293 with *AtPQL1a* in 10 mM K^+^ and 0.05m Ca^2+^ (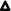) or 10 mM K^+^ an 2 mM Ca^2+^ (▲)

In order to investigate further the transport properties of *AtPQL1a* and *HvPQL1*, the patch clamp assay was performed using HEK293 cells. When measuring currents in whole‐cell configuration from a non‐transfected HEK293 cell, the only detectable current in Na^+^‐containing bath solutions was an *I*_A_‐like current that has an initial fast activation followed by a slower inactivation. This current rectifies outwardly and was activated by depolarization steps. No additional currents could be resolved when hyperpolarizing the cells (Figure 3a). On the other hand, large currents were recorded from HEK293 cells transfected with either *AtPQL1a* or *HvPQL1* (Figure 3). By examining the instantaneous currents as a function of voltage, we observed a linear relationship lacking any rectification (Figure 3b) with an apparent reversal potential (*E*_rev_) close to −35 mV. As the Na^+^ concentrations in the external solution were increased, the reversal potential (*E*_rev_) shifted to the less negative values (Figure 3b). Absolute values for E_rev_ varied between experiments, this was likely due to differences between HEK293 cells in the size of the background currents, and thus effects of those current on overall *E*_rev_.

*HvPQL1* discriminated poorly between monovalent cations such as K^+^, Na^+^, and Li^+^, with Li^+^ showing slightly higher permeability relative to both Na^+^ and K^+^ (Figure 3c). The inward currents were inhibited by addition of high external calcium (Figure 3c) and marginally inhibited by external acidification (Figure 3d), two notable properties of Na^+^ influx currents in plants. The electrical currents facilitated by *AtPQL1a* (Figure 3e) are similar to currents observed for *HvPQL1*, and the currents facilitated by *AtPQL1a* is more sensitive to inhibition by external calcium compared to *HvPQL1*.

### 3.4 *PQLs* localize into the internal membrane structures *in planta*

**FIGURE 4:**
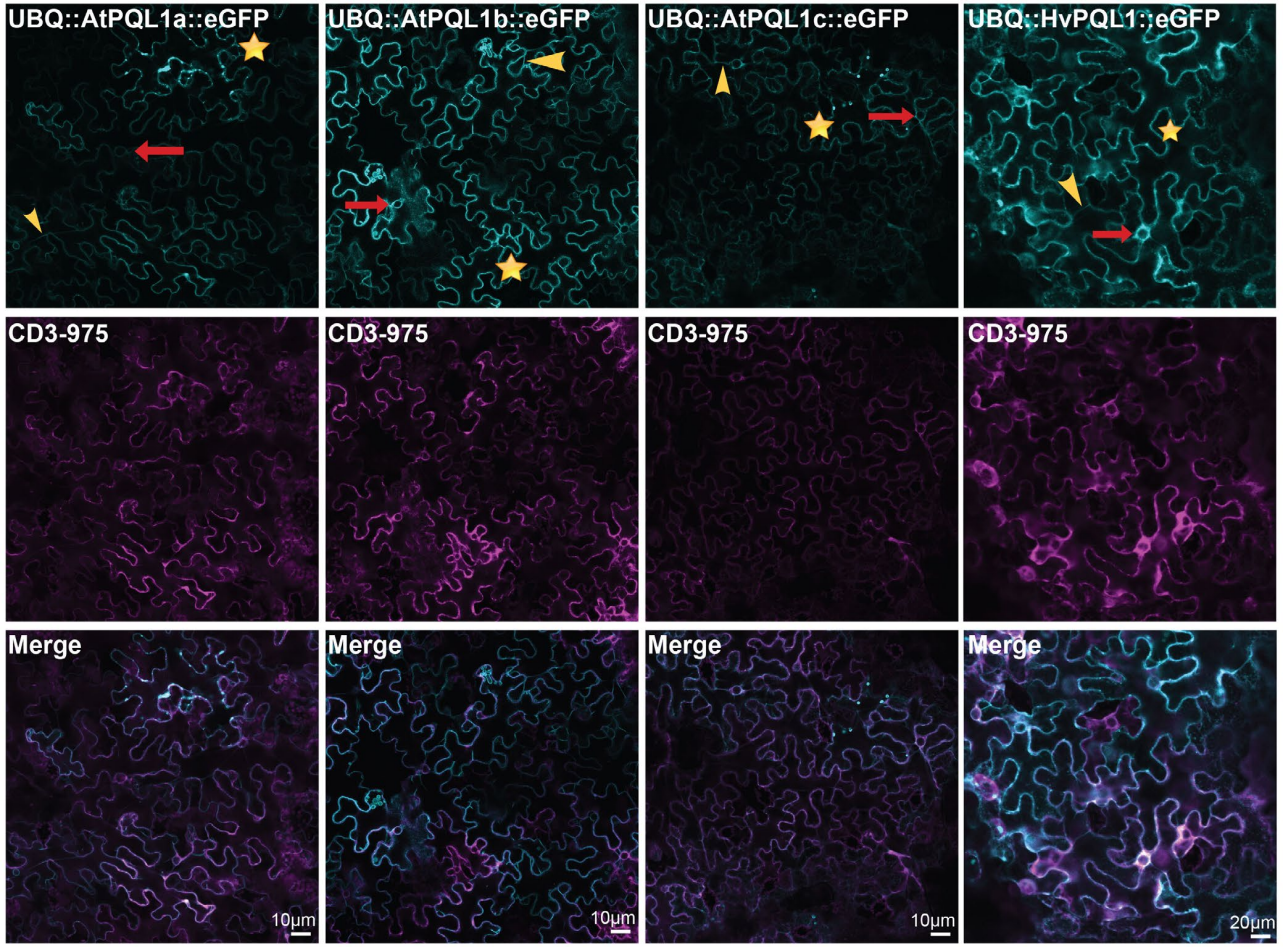
AtPQL1a, AtPQL1b, AtPQL1c, and HvPQL1 co‐localize with the tonoplast marker during transient co‐expression in N. benthamiana leaf epidermal cells. Transient expression of UBQ10p::AtPQL1a‐eGFP, UBQ10p::AtPQL1b‐eGFP, UBQ10p::eGFP‐AtPQL1c, and UBQ10p::HvPQL1‐eGFP was observed using confocal laser scanning microscopy. For all transformants, the PQL proteins (left panel) were co‐infiltrated with tonoplast marker (CD3‐975::*mCherry*, middle panel), and the overlay of the two was used to study co‐localization. The red arrows indicate the circular structures (bulbs) that formed in the lumen of the vacuole. The yellow stars indicate the separation between the two neighboring cells. The yellow arrowheads represent the transvacuolar strands. The images are representative of 15 replicates, visualized during 3 independent microscopy sessions

In order to examine the subcellular localization of PQL proteins *in planta*, the subcellular localization of *AtPQL1a*, *AtPQL1b*, *AtPQL1c,* and *HvPQL1* fused to GFP at the C‐terminal end was performed using transient transformation of *N. benthamiana* epidermal cells and stable transformation of Arabidopsis. The tonoplast marker (*CD3‐975*::*mCherry*) was co‐expressed with *AtPQL1a*, *AtPQL1b*, *AtPQL1c,* and *HvPQL1* (Figure 4). For all the studied plant PQL constructs, we observed the circular structures, which are typical for the lumen of the vacuole in these highly vacuolated cells. Additionally, we observed a clear separation between the neighboring cells, which is an indicator that the membrane structures to which PQLs localize are not interacting, and thus suggest an internal membrane localization, rather than plasma membrane. Furthermore, PQL localized to transvacuolar strands, resulting from the vacuole‐forming cytoplasmic tunnels, were observed (Figure 4, Figure S3), providing an additional indication for PQL localization to the tonoplast membrane. Additionally, when the subcellular localization of *AtPQL1a*, *AtPQL1b*, *AtPQL1c,* and *HvPQL1* was overlaid with the tonoplast marker (*CD3*‐*975*::*mCherry*), we observed a co‐localization, providing further evidence that the internal membrane structures to which PQL localizes might be to the vacuolar membrane (Figure 4).

**FIGURE 5:**
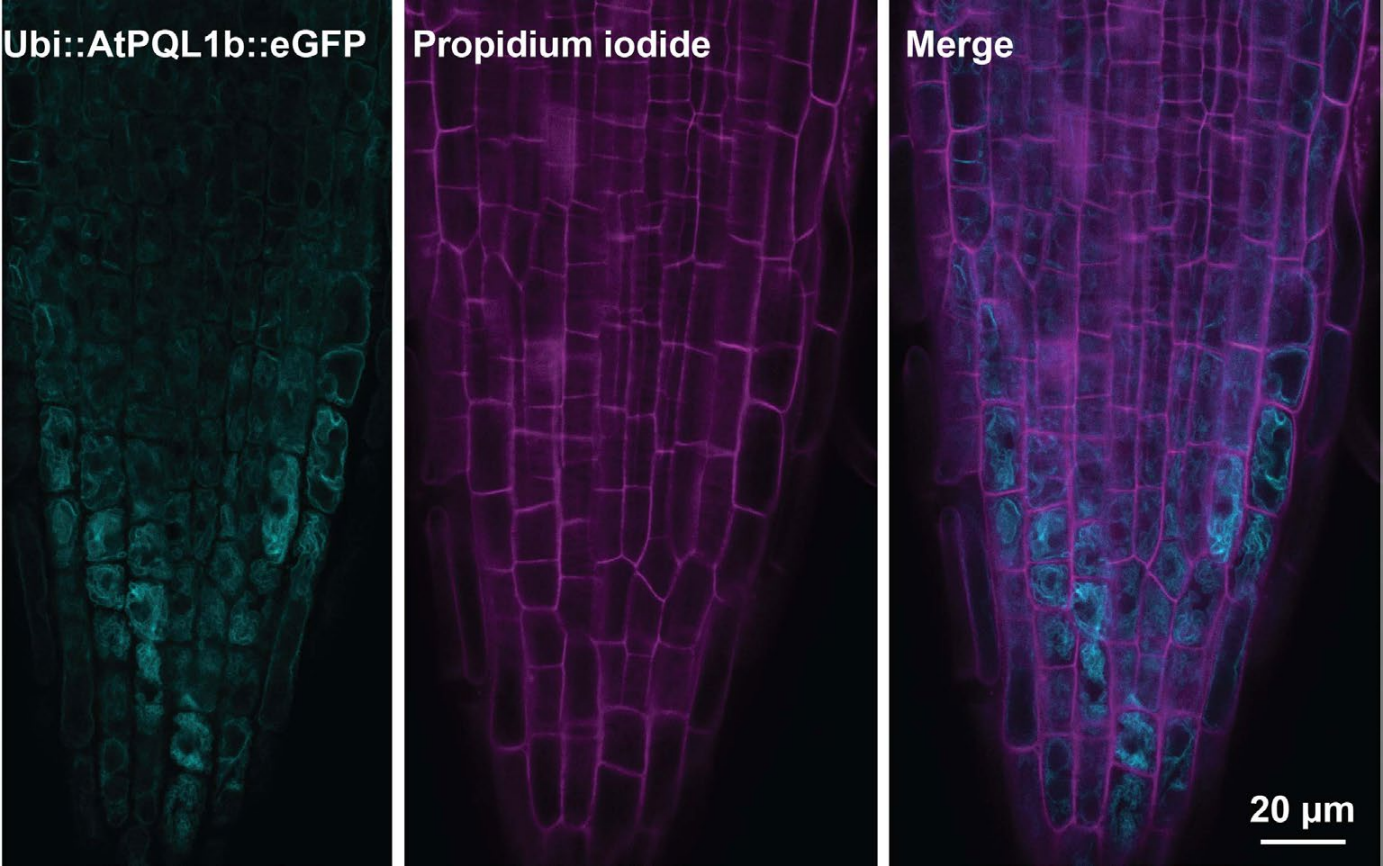
*AtPQL1b* localizes in the internal membrane compartments in stably transformed Arabidopsis root tips. The root tips of stably transformed Arabidopsis plants were imaged using confocal laser scanning microscopy. The left panel shows the root epidermal cells stably transformed with *UBQ10p::AtPQL1b‐GFP*. The middle panel shows the propidium iodide staining of the same cells, while the right panel is the merge between the two channels. Similar results were observed in 12 replicates, visualized during 3 independent microscopy sessions

As the *N. benthamiana* epidermal cells are heavily vacuolated, we also studied the localization of *AtPQL1b* in the root tip of stably transformed Arabidopsis Col‐0 plants using *UBQ10p::AtPQL1b::GFP* construct. Confocal images of the root tips indicated that *AtPQL1b* is localized to internal membrane compartments (Figure 5), as their localization did not overlap with the propidium iodide stain, which is indicating the cell walls. The vacuoles closer to the root tip are very fragmented, and the fragmentation decreases in the more differentiated parts of the root. We observed that the pattern for *AtPQL1b* was similar to the patterns observed for vacuolar fragmentation along the developmental axis, with higher fragmentation toward the tip of the root, and more uniform compartments in the elongation and differentiation zones. Summarizing, we observed that clade 1 PQLs localize to internal membrane structures. Based on the morphological characteristics typical to the vacuoles, and co‐localization with the tonoplast marker, the most probable localization of the plant PQLs is the vacuolar membrane.

### 3.5 Expression of clade 1 *PQLs* in plants is responsive to salinity stress and nutrient deprivation

**FIGURE 6:**
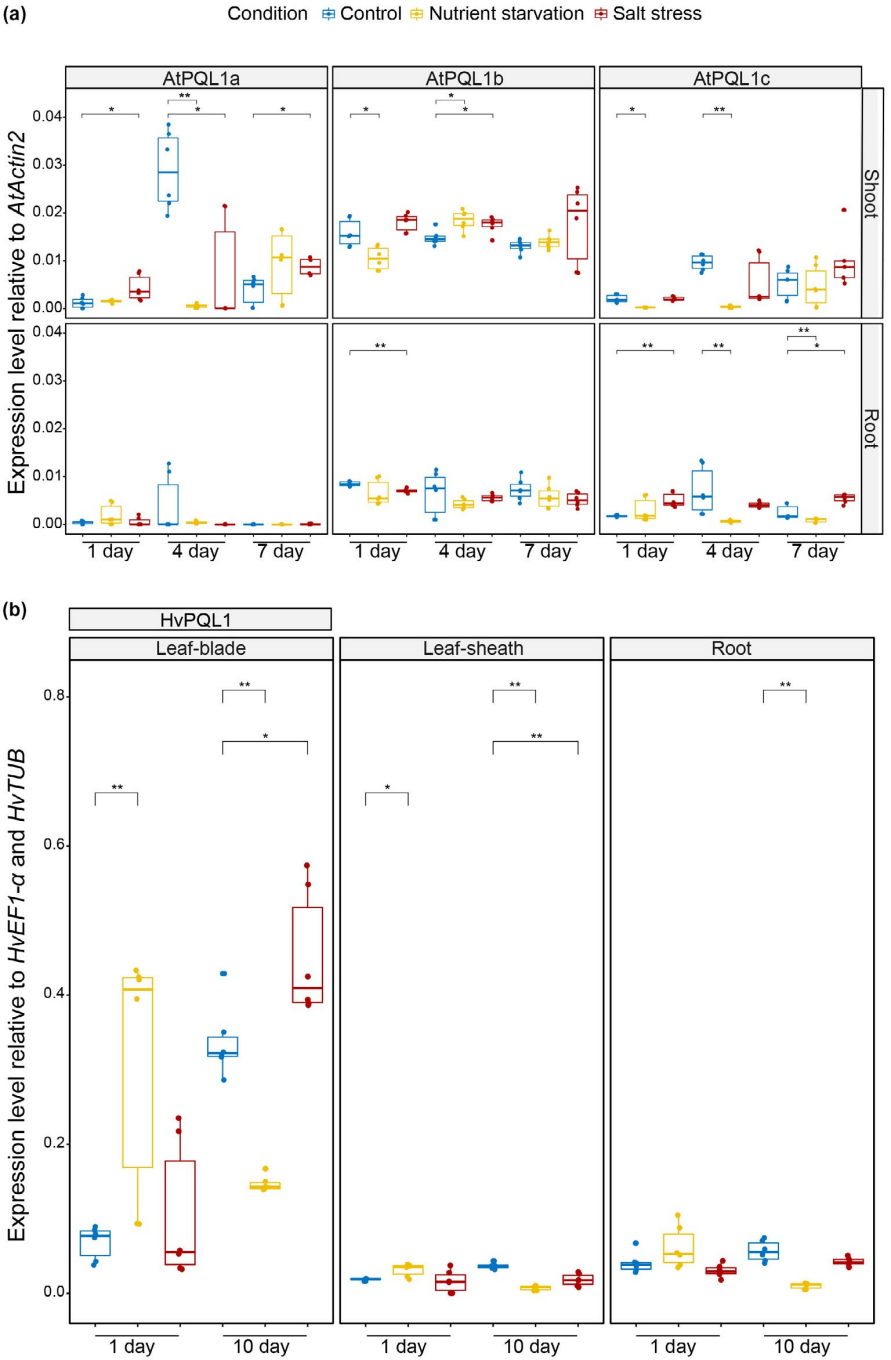
Transcripts of Arabidopsis and barley PQLs are responsive to environmental stress. (a) Relative expression level of AtPQL1a, AtPQL1b, and AtPQL1c in shoot and root tissues of Arabidopsis grown under control (blue), salt stressed (100 mM NaCl; red), and nutrient starved (0.1 mM CaCl2; yellow) conditions. Three‐week‐old plants were used to apply the treatment and the samples for transcript level estimation were collected 1, 4, or 7 days after exposure to stress conditions. (b) The expression level of HvPQL1 was examined in leaf‐blade, leaf‐sheath, and root tissues of plants grown under control (blue), salt stressed (100 mM NaCl, red), and nutrient starved (0.1 mM CaCl2, yellow) conditions. Two‐week‐old plants were exposed to stress and the samples for transcript level estimation were collected 1 or 10 days after the exposure to stress conditions. The box plots represent the median expression value based on six biological replicates. The boxes represent the 1.5*Interquartile Range. Statistically significant differences between control and other stress conditions are indicated as * (*p* < .05) and ** (*p* < .01) as determined using ANOVA with pairwise Tukey HSD test

As NSCCs are suggested to play an important role in ion transport, in particular of Na^+^ (Munns & Tester, 2008), it is reasonable to hypothesize that they could play a role in salinity and nutrient stress. Therefore, we examined tissue‐specific expression of the clade 1 *PQLs* in *Arabidopsis thaliana* and barley (v. Morex; Figure 6). We observed that 1 and 7 days after application of salt stress treatment, the expression of *AtPQL1a* was upregulated compared to that seen in control conditions. This upregulation was specific to shoot tissue (Figure 6a). The expression of *AtPQL1b* was also upregulated in response to salinity stress in shoot tissue, but the difference was found to be significant only 4 days after stress application (Figure 6a). The root‐specific downregulation of AtPQL1b in response to salt stress was significant only 1 day after stress application. The salt‐induced changes in expression of *AtPQL1c*, on the other hand, were only observed in the root tissue after 1 and 7 days of salt stress (Figure 6a). Nutrient starvation (0.1 mM CaCl_2_ solution without other nutrients) also affected the expression levels of Arabidopsis clade 1 PQLs. The transcript levels of *AtPQL1b* were upregulated 4 days after stress application in the shoot. Interestingly, the levels of AtPQL1c transcripts were reduced, first in the shoot at 1 and 4 days after nutrient deprivation, and subsequently in the root, 4 and 7 days of nutrient deprivation (Figure 6a). In summary, *AtPQL1a* and *AtPQL1b* are transcriptionally activated in response to salinity stress, and most of the responses are confined to the shoot tissue. The *AtPQL1c*, on the other hand, is transcriptionally repressed in response to nutrient deprivation, and this is starting at the shoot level followed by downregulation in the root.

The primary site of *HvPQL1* expression was observed in the leaf‐blade, with its expression also responsive to nutrient deprivation and salt stress. Similar to *AtPQL1a* and *AtPQL1b* expression, the expression of *HvPQL1* was upregulated in response to salt in in leaf‐blade 10 days after imposition of salt stress (Figure 6b). Interestingly, in response to nutrient deprivation, the expression of *HvPQL1* was first upregulated in leaf‐blade and leaf‐sheath, and subsequently repressed in all studied tissues 10 days after exposure to nutrient deprivation (Figure 6b). These results suggest that the expression of HvPQL1 is altered in response to salt stress and nutrient deprivation, similar to the three PQL1 homologues in Arabidopsis.

### 3.6 Single *AtPQL1* clade 1 mutants showed slightly enhanced salt stress tolerance

**FIGURE 7:**
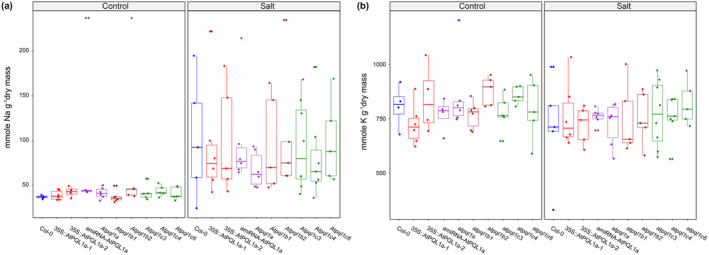
The Arabidopsis PQLs clade 1 does not significantly affect the ion accumulation. Two overexpression lines of AtPQL1a (35S::AtPQL1a‐1 and 35S::AtPQL1a‐2) and knock‐down lines of *AtPQL1a*, *AtPQL1b*, *AtPQL1c* (amiRNA‐AtPQL1a, *Atpql1a, Atpql1b1*, *Atpql1b2*, *Atpql1c3, Atpql1c4, Atpql1c6*), and Col‐0 were germinated under control conditions. Salt stress (~80 mM NaCl) was applied at 17 days after germination, and the ion content (Na^+^, K^+^), fresh and dry mass were determined 7 days after stress application for 5 replicates per condition per genotype. The bars represent the median of 5 biological replicates. The boxes represent the 1.5*Interquartile Range. The differences between Col‐0 and individual mutant lines were tested using a *t*‐test. No significant differences were observed

**FIGURE 8:**
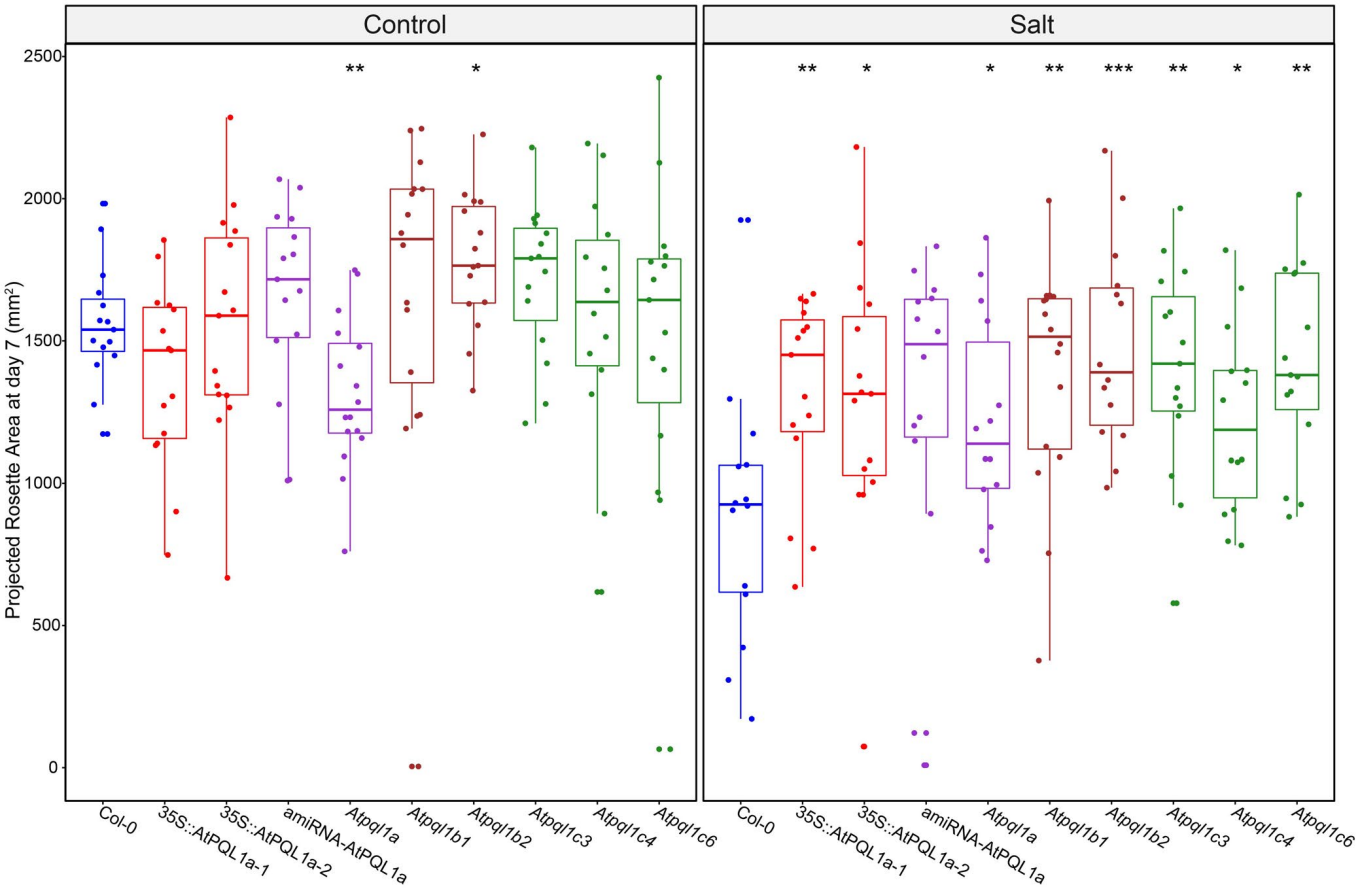
Disruption of clade 1 PQL function results in larger plants under salt stress conditions. The Projected Rosette Area estimated 7 days after application of treatment for Col‐0, 35S::AtPQL1a‐1, 35S::AtPQL1a‐2, amiRNA‐AtPQL1a, *Atpql1a*, *Atpql1b1*, *Atpql1b2*, *Atpql1c3, Atpql1c4,* and *Atpql1c6*. Seventeen‐day‐old plants were exposed to treatment (control or ~ 80 mM NaCl) and their Projected Rosette Area was scored using automated phenotyping system (PSI, Czech Republic) from the RGB images. Box plots represent the median of 15 replicates per genotype and treatment. The boxes represent 1.5*Interquartile Range. The significant differences between Col‐0 and the other genotypes per condition are indicated with *, **, or *** for *p*‐values below .05, .01, and .001, respectively, as calculated using *t*‐test

NSCC are hypothesized to be the facilitator of the sodium ion entry during salt stress and are thus likely to play a role in salinity tolerance. To study whether the alteration of clade 1 PQL levels affects salt stress tolerance, we examined Arabidopsis lines with increased (35S::AtPQL1a‐1, 35S::AtPQL1a‐2) or decreased (amiRNA‐AtPQL1a, *Atpql1a*, *Atpql1b1*, *Atpql1b2*, *Atpql1c3*, *Atpql1c4*, and *Atpql1c6*) levels of clade 1 PQLs levels. We examined ion accumulation, plant growth, rosette color, and photosynthetic performance over time under control and salt stress conditions in soil‐grown plants. No significant differences were observed between Col‐0 and mutant lines for accumulation of sodium or potassium ions measured at the end of the experiment (Figure 7). While we did not observe any significant differences between the studied lines in non‐stress conditions, all lines with altered PQL1 levels grew larger rosettes after 7 days of salt stress exposure (Figure 8). Based on the projected rosette area, we estimated the Relative Growth Rate (RGR) for individual plants by fitting an exponential function (e ^ {RGR x time(d) + Intercept}) to area over time (Figure S2). While almost all mutant lines were observed to have a higher projected rosette area than Col‐0 in salt stress conditions at the last day of measurement (Figure 8), the no differences in RGR were observed between the genotypes (Figure S2). This observation could be caused by developmental effects of PQL1, although we did not observe any differences between PQL1 mutant lines and Col‐0 grown under control conditions (Figure 8, Figure S2).

The effect of salt stress on the photosynthetic performance was assessed daily at different photon irradiances using chlorophyll fluorescence images, using the light curve protocol (Henley, 1993). The significant differences between Col‐0 and some mutant lines were observed for maximal fluorescence for dark‐adapted state (F_m_), variable fluorescence for dark‐adapted state (F_v_), instantaneous fluorescence level (F_t_), and photochemical quenching (F_q_) under control and salt stress conditions at the last day of experiment (Figure S4).

In summary, the above results suggest that modifications in the clade 1 PQLs levels, either through overexpression or knock‐down of the PQL genes, result in larger plant size and improved photosynthetic performance at the later stages of salt stress exposure compared to the wild‐type plants.

### 3.7 *AtPQL1a* and *AtPQL1b* affect development of lateral roots under control and ionic stress conditions

Although the expression of the clade 1 PQLs is predominant in the shoot tissue, the PQLs could potentially play an important role in root system architecture, contributing to the ion compartmentalization into the vacuole in concert with other proteins. Therefore, we studied the Root System Architecture phenotypes of PQL mutants and compared them to Col‐0. Four major Root System Architecture traits were analyzed: main root length, total lateral root length, lateral root number, and total root length.

**FIGURE 9:**
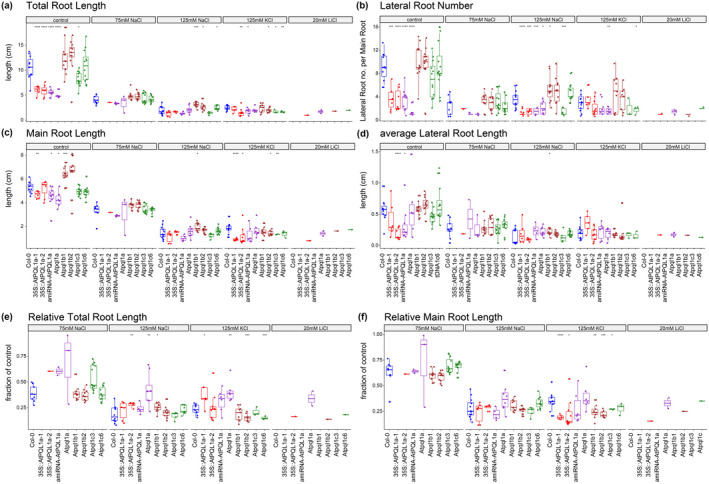
AtPQL1a and AtPQL1b affect the development of lateral roots under control conditions. Four‐day‐old seedlings were transferred from 0 mM NaCl to 75, 125 mM NaCl, 125 mM KCl, and 20 mM LiCl. RSA was quantified in 12 replicates after 6 days of treatment. The box plots represent the distribution of the samples for (a) the total root size (cm), (b) Lateral Root Number (#LR / MR), (c) Main Root Length (cm), (d) average Lateral Root Length, as well as (e) the relative Total Root Length (fraction of average Total Root Length at 0 mM NaCl) and (f) the relative Main Root Length (fraction of average Main Root Length at 0 mM NaCl). The significant differences between the Col‐0 and studied mutant lines are indicated by *, **, and *** for *p*‐value below .05, .01, and .001 per treatment and calculated using *t*‐test

The lines with increased or decreased expression of AtPQL1a (35S::AtPQL1a‐1, 35S::AtPQL1a‐2, amiRNA‐AtPQL1a, *Atpql1a*) were severely impaired in root development, mainly due to reduced lateral root development (Figure 9a,b). The plants lacking functional AtPQL1b (*Atpql1b1* and *Atpql1b2*), on the other hand, grew longer main roots, as well as longer and more lateral roots compared to Col‐0 under non‐stress conditions (Figure 9b,d). The lines lacking functional AtPQL1c (*Atpql1c‐3*, *Atpql1c‐6*) were not affected in the main root development (Figure 9c), but the individual lines showed opposing phenotypes in their lateral root development (Figure 9b,d). These developmental differences between clade 1 PQL mutants and Col‐0 were further maintained under salt stress conditions (75 and 125 mM NaCl), while positive effect of AtPQL1b mutations on root development was not evident when the seedlings were grown on medium supplemented with either KCl or LiCl (Figure 9).

When normalized for developmental differences, the performance of AtPQL1a lines (35S::AtPQL1a‐1, 35S::AtPQL1a‐2, amiRNA::AtPQL1a, and *Atpql1a*) was only further compromised when the seedlings were grown on plates supplemented with LiCl (Figure 9e,f). The relatively better performance of AtPQL1b lines, on the other hand, was cancelled under salt stress conditions, and the AtPQL1b mutants were actually performing worse when grown on LiCl plates. The AtPQL1c mutants (*Atpql1c3* and *Atpql1c6*) also showed decreased performance on LiCl media compared to Col‐0, but the effect was not as severe as for AtPQL1a mutants. These results suggest that Arabidopsis clade 1 PQLs play an important role in root development under control conditions and affect plant performance under ionic stress conditions, especially when exposed to LiCl.

## 4 DISCUSSION

Salinity tolerance is determined, in part, by ion transport, which encompasses the initial flux of ions into the root and compartmentalization of ions into non‐photosynthetic tissues and/or vacuoles (Volkov & Beilby, 2017). Non‐selective cation channels were previously shown to be important for Na^+^ influx into root cells (Amtmann & Sanders, 1998; Davenport & Tester, 2000; Demidchik et al., 2002; Demidchik & Maathuis, 2007; White, 1999), yet their genetic constituents are yet to be identified. In this study, we identified plant homologues of yeast PQLs (Figure 1), and identified clade 1 to contain the closest homologues of yeast PQLs, previously described to mediate the flux of several cations, including K^+^ and Na^+^, in yeast (Carter, 2009). We performed functional characterization of clade 1 PQL from Arabidopsis and barley, including ion transport characteristics and their role in plant development, growth, and salt tolerance.

We observed that AtPQL1a and HvPQL1 are involved in the transport of monovalent cations, such as Na^+^, K^+^, and Li^+^ (Figures 2, 3). The PQL1 permeability was inhibited by high external Ca^2+^ and pH acidification (Figure 3c,d), which is also typical for previously described Ca^2+^‐sensitive voltage‐insensitive non‐selective cation channels (vi‐NSCCs) reported for root‐derived protoplasts from several plant species (Buschmann et al., 2000; Demidchik & Tester, 2002; Spalding et al., 1992; Tyerman et al., 1997). Only one difference was noticed, which is vi‐NSCC favored K^+^ over Na^+^, while in clade 1 PQLs showed higher permeability to Li^+^ than K^+^ and Na^+^ (Figure 3c). The characteristics of clade 1 PQLs were previously reported to be similar in their transport properties to vi‐NSCC (Carter, 2009; Shearer, 2013). As vi‐NSCC is considered to be the major pathway for Na^+^ influx into the plant root (Amtmann & Sanders, 1998; Davenport & Tester, 2000; Demidchik et al., 2002; Demidchik & Maathuis, 2007; White, 1999), we initially hypothesized that clade 1 PQLs were located to the plasma membrane of plant cells.

However, results presented in this study suggest that the clade 1 PQLs in Arabidopsis and barley are localized to internal membrane compartments, most likely the tonoplast (Figures 4, 5). The NSCCs that have been reported to be in the tonoplast were either slow (SV) or fast (FV) vacuolar channels (Demidchik et al., 2002; Pottosin & Dobrovinskaya, 2014). SV cation channels are activated by Ca^2+^ and voltage (Demidchik et al., 2002; Pottosin & Dobrovinskaya, 2014), which is different from the transport properties of clade 1 PQLs (Figure 3). Clade 1 PQLs are also unlikely to belong to non‐selective voltage‐dependent FV cation channels, as the activity of PQLs is not voltage dependent. While both FV channels and clade 1 PQLs channels are inhibited by increased non‐cytosolic Ca^2+^, the FVs are also inhibited by cytosolic Ca^2+^ and Mg^2+^ (Demidchik et al., 2002), which were not tested in this study. Additionally, clade 1 PQLs showed slight inhibition in response to low pH, while the same range of pH was not tested for FV (Demidchik et al., 2002).

In summary, AtPQL1a and HvPQL1 are Ca^2+^‐sensitive, voltage‐insensitive NSCC located to the vacuolar membrane. Further experiments will reveal the role of PQLs in ion transport and their relationship to other known NSCCs such as the FV channels.

We did not observe any changes in ion accumulation (Figure 7), unlike for the mutants of other ion transporters, including *sos1* and *hkt1*, where increased accumulation of Na^+^ ions under salt stress conditions was observed (Ji et al., 2013; Møller et al., 2009; Wu et al., 1996; Yang et al., 2009). However, SOS1 and HKT1 have presumably high specificity for Na^+^ over other transported ions (Møller et al., 2009), which is unlike the PQLs, which may provide some explanation for no significant differences observed in our study. Interestingly, we found that clade 1 PQLs play an important role in lateral root development (Figure 9). Some of the ion transporters were earlier described to affect plant development, including NHX (Bassil et al., 2011) and HKT1 (Julkowska et al., 2017). The effect of the clade 1 PQLs on root development is clearly visible in the non‐stress conditions (Figure 9). Whether the single mutations in the clade 1 PQLs affect lateral root development by affecting vacuolar function remains to be studied.

Interestingly, we did not observe any developmental phenotypes related to the PQL mutants under control conditions when the plants were grown in the soil for a long‐term salt stress experiment (Figure 8, Figures S4). This could be due to a compensatory mechanism activated over the longer term. Additionally, as salt stress affected the expression of *AtPQL1a* and *AtPQL1b* (Figure 6), these two genes might act redundantly. Studying the multiple knock‐out of clade 1 PQLs or knock‐out mutants of *HvPQL1* in barley might provide more insight in the role of PQL in salt stress tolerance as well as developmental processes.

This study is a first step toward the understanding of the function of the plant homologues of yeast *ScPQL* proteins. We present evidence that plant clade 1 PQLs transport monovalent cations in a non‐specific fashion are localized to internal membranes and affect development of lateral roots in Arabidopsis seedlings.

## Supporting information

Supplementary Material
