# Supplementary material for: The role of PQL genes in response to salinity tolerance in Arabidopsis and barley
Source: Plant Direct. 2021 Feb 10;5(2):e00301. doi: 10.1002/pld3.301 (PMC7876507; doi:10.1002/pld3.301)
Supplement: Supplementary file 1 — Supplementary Material [file PLD3-5-e00301-s001.docx]

**
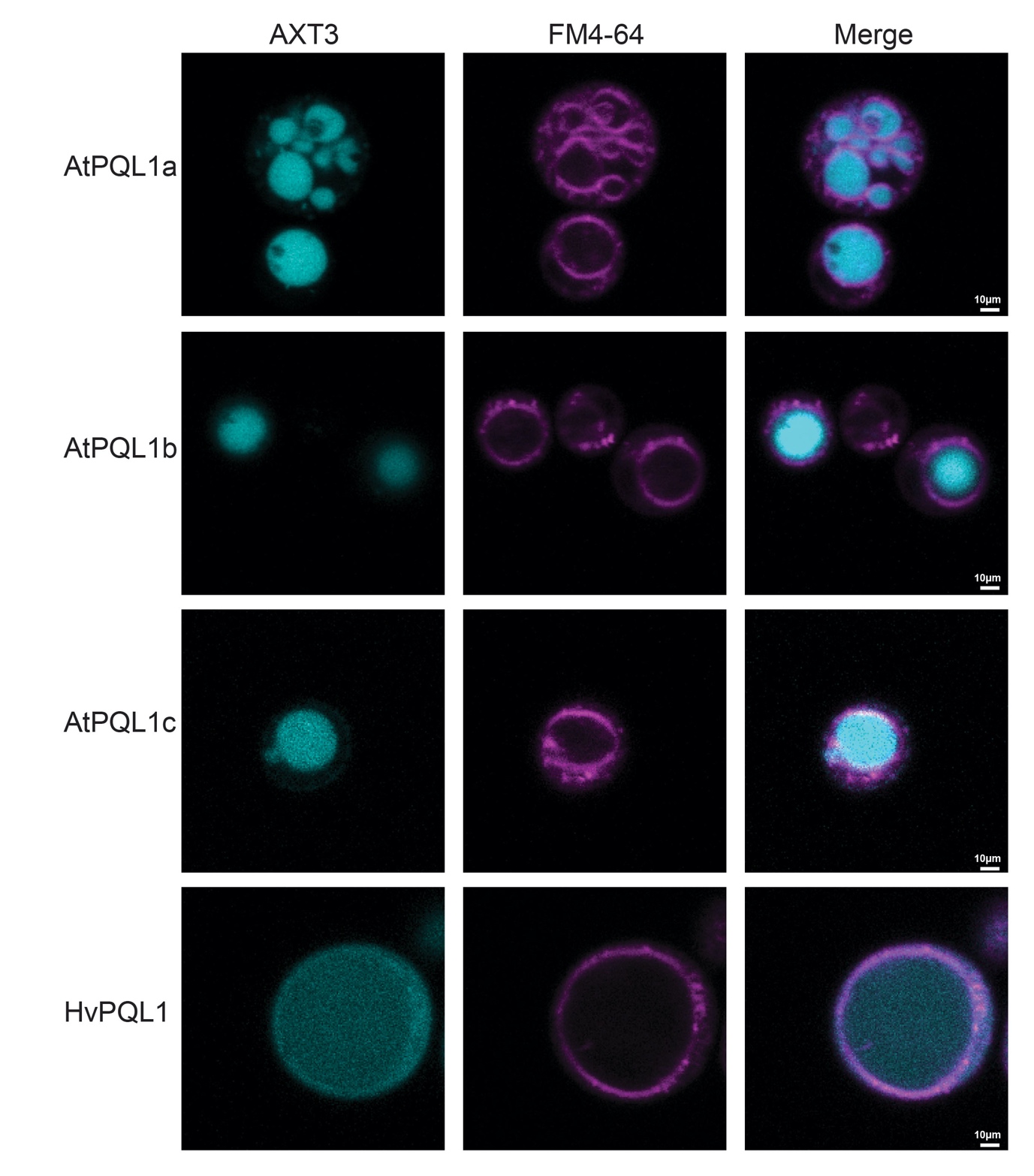
**

Figure S1. The sub-cellular localization of AtPQL1a, AtPQL1b, AtPQL1c and HvPQL1 was examined in AXT3 strain carrying PQL proteins fused to eGFP at the C-terminus. The detected GFP-signal is presented in the left panel, while membrane stain (FM4-64) was detected in the red-channel, presented in the middle panel. The right panel presented the overlap between FM4-64 and GFP signal. The images are representative of at least 9 replicates, visualized during 3 independent microscopy sessions. Each session consisting of 3 independent transformations per construct. The scale bar represents 10µm.

**
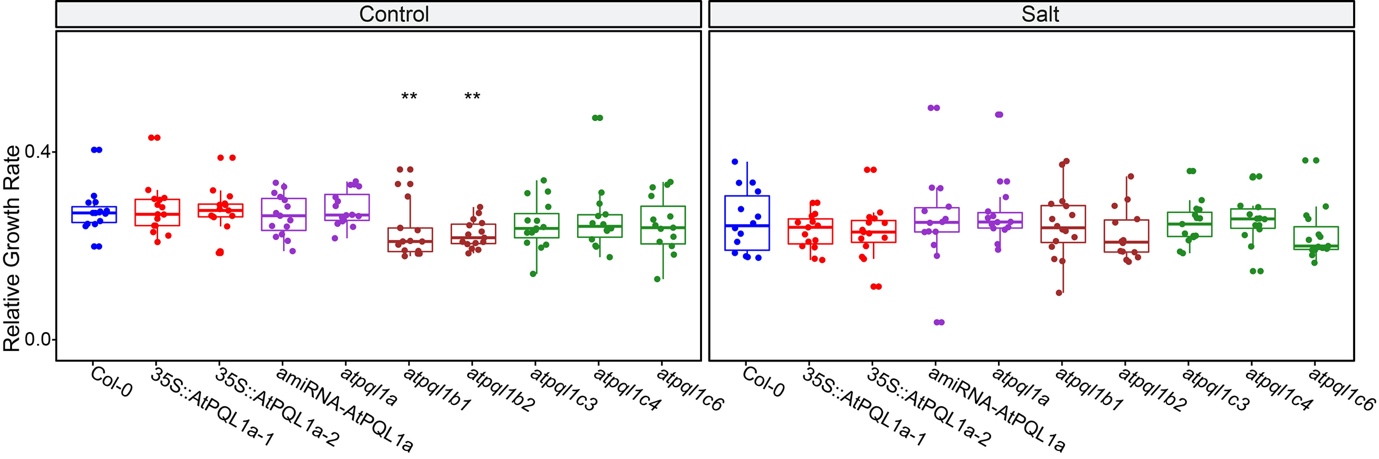
**

**Figure S2.** **Single mutants of clade one PQLs do not show differences in Relative Growth Rate.** Relative growth rate (RGR) of Col-0, 35S::AtPQL1a-1, 35S::AtPQL1a-2, amiRNA-AtPQL1a, *Atpql1a*, *Atpql1b1*, *Atpql1b2*, *Atpql1c3*, *Atpql1c4* and *Atpql1c6*, was estimated by fitting the exponential function (Area=e^Delta x time (days) + Intercept^, where Delta is RGR) to the increase in Projected Rosette Area of individual plant. The boxes represent the 1.5*Interquartile Range. The significant differences between Col-0 and the other genotypes per condition are indicated with * or ** or *** for p-values below 0.05 and 0.01, respectively, as calculated using t-test.

**
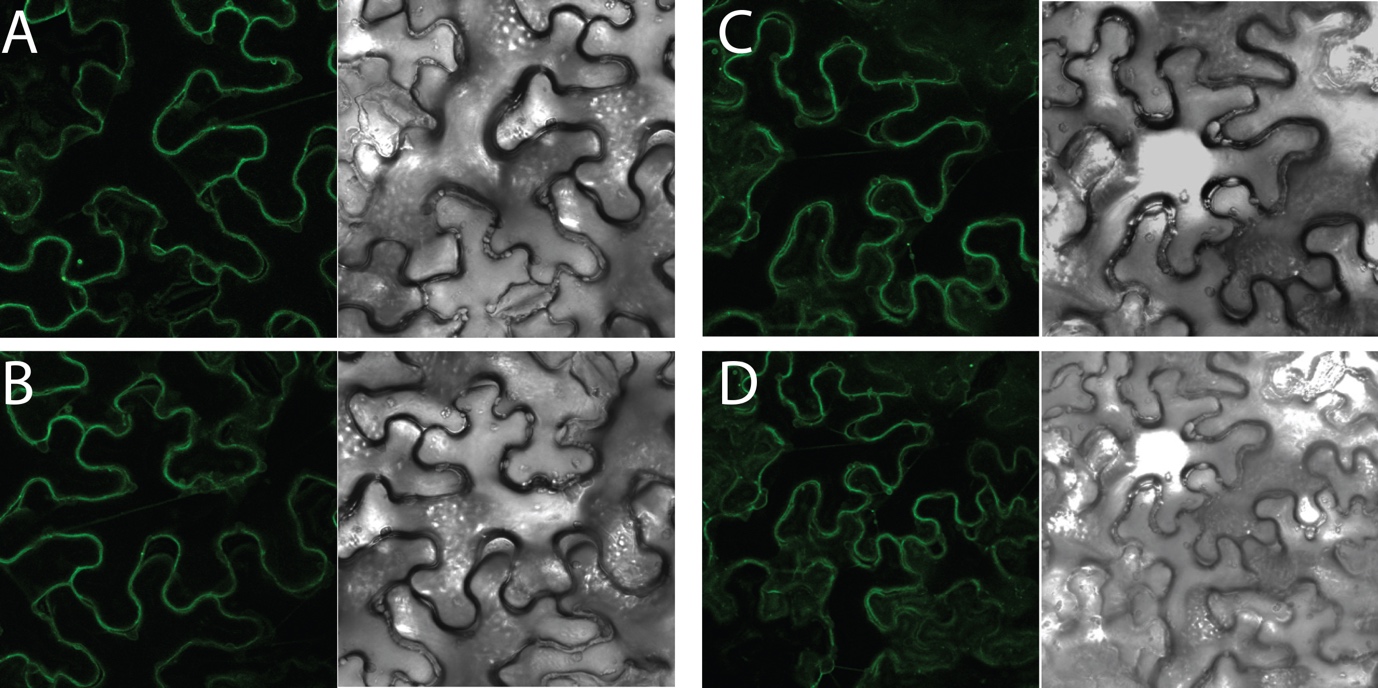
**

**Figure S3.** **AtPQL1b localizes into vacuole specific patterns.** Transient expression of UBQ10p::eGFP-AtPQL1c was observed using confocal laser scanning microscopy. For all transformants, the PQL proteins (left panel) were infiltrated, and their localization was compared to the bright-field picture taken at the same focal plane (right panel). The circular structures (bulbs) that formed in the lumen of the vacuole, separation between the two neighboring cells and transvacuolar strands can be observed at four (**A-D**) representative instances. The images are representative of 15 replicates, visualized during 3 independent microscopy sessions.

**
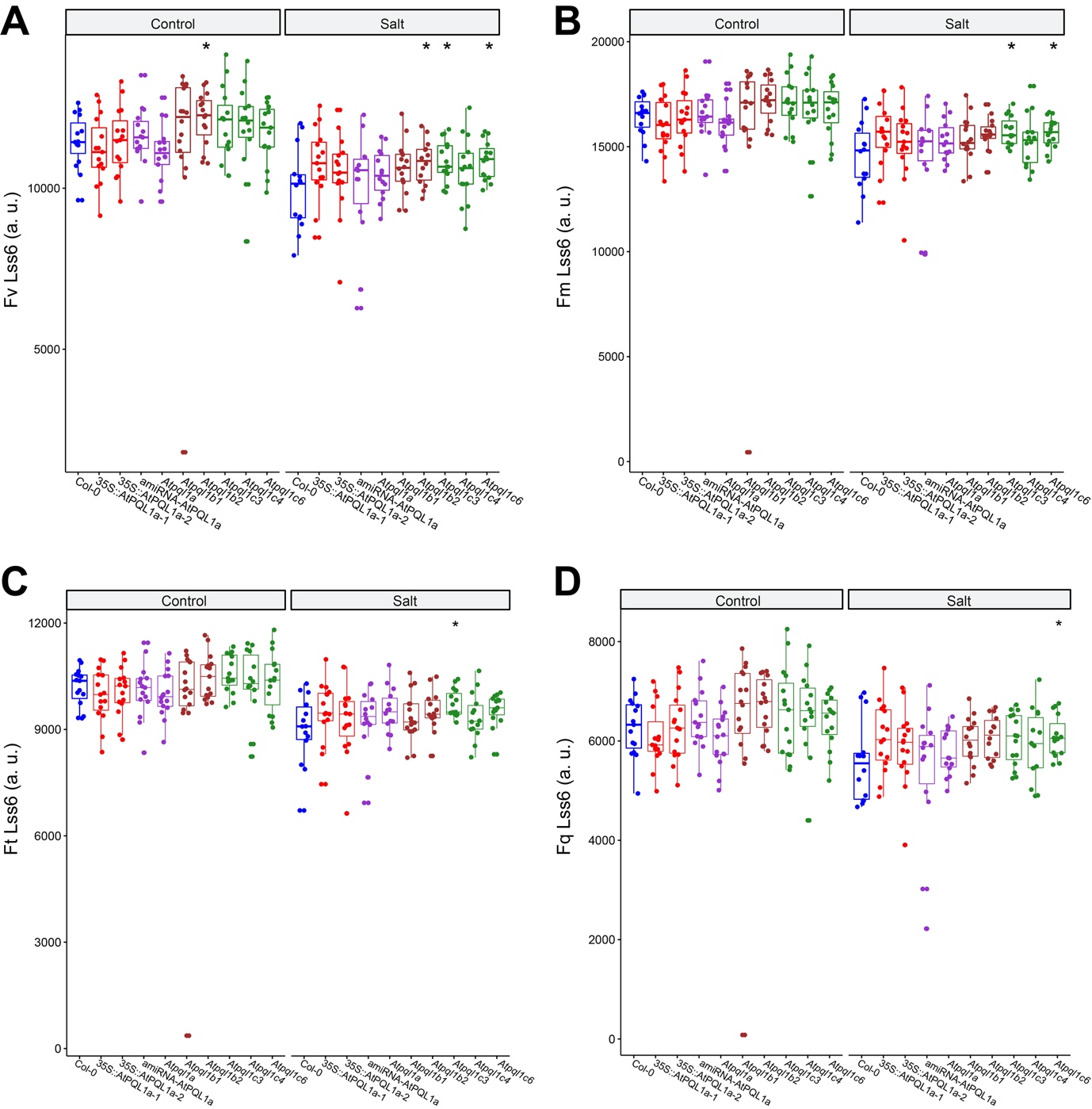
**

**Figure S4**. **The effect of salt stress on ChlF parameters**. **(A)** Variable fluorescence of PSII for the dark-adapted state (F_v_), **(B)** the Maximal fluorescence of PSII for the dark-adapted state (F_m_), **(C)** Instantaneous fluorescence level at time t (F_t_), **(D)** a measurement for the effective photosynthesis for plants in the dark (F_q_) were measured using the light curve protocol for Col-0 and the mutant lines in control and salt stress (~80 mM NaCl) conditions in the last day of the experiment (day 7). The boxes represent the 1.5*Interquartile Range. The significant differences between Col-0 and the other genotypes per condition are indicated with * for p-values below 0.05 as calculated using t-test.

**Table S1. Primers used in the yeast and co-localization CDS cloning**

|  | **Primer name** | **Primer sequence (5'-3')** |
| --- | --- | --- |
| AtPQL1a | AtPQL1a.f | **CACC**ATGATTCGAGATGATTT |
|  | AtPQL1a.r | GACAGCTTCTTCACCGGTTT |
| AtPQL1b | AtPQL1b.f | **CACC**ATGTTTCTCCACAGCAG |
|  | AtPQL1b.r | GACAGCTTCTGCGGTTTCAT |
| AtPQL1c | AtPQL1c.f | **CACC**ATGGTGTCTTTAGGTTACT |
|  | AtPQL1c.r | CGAAACAAAAGTTTTGCTTG |
| HvPQL1 | HvPQL1.f | **CACC**ATGGGTATCTTCAGTGG |
|  | HvPQL1.r | GGCTTTATCCAAATTGTCT |

|  | **Primer name** | **Primer sequence (5'-3')** | **Expected amplicon size** |
| --- | --- | --- | --- |
| HvEF1-α  (AK252297) | HvEF1a.f | ATGATTCCCACCAAGCCCAT | 101 bp |
|  | HvEF1a.r | ACACCAACAGCCACAGTTTGC |  |
| HvtubA (U40042) | HvTUB.f | AGTGTCCTGTCCACCCACTC | 247 bp |
|  | HvTUB.r | AGCATGAAGTGGATCCTTGG |  |
| HvPQL1  ( AK361243 ) | HvPQL1.f | GGGCTCAGTCCGCTAATGTT | 125 bp |
|  | HvPQL1.r | TCCAGCATCTACAAGCCACG |  |
| AtActin2  (At3g18780) | AtACT2.f | GGTAACATTGTGCTCAGTGGTGG | 107 bp |
|  | AtACT2.r | AACGACCTTAATCTTCATGCTGC |  |
| AtPQL1a  (At4g20100) | AtPQL1a.f | GAGGGAAAGATAGAGTGTTTGTAG | 183 bp |
|  | AtPQL1a.r | CCCTCAACATTTCCTCTCCTTAC |  |
| AtPQL1b  (At2G41050) | AtPQL1b.f | ATGTCACGCATCCTTCCACC | 181 bp |
|  | AtPQL1b.r | TGCTACGTTGCTGCTAGTGA |  |
| AtPQL1c  (At4G36850) | AtPQL1c.f | GGCTAGTCTTCCGCTTCAGG | 125 bp |
|  | AtPQL1c.r | GCCATTAGCCATCCCAACCA |  |

Table S2. Primers used in the q-RT-PCR experiment.
